# Organic Phototransistor Photonic Synapses for Artificial Vision

**DOI:** 10.1007/s40820-025-02036-0

**Published:** 2026-01-12

**Authors:** Feng Ding, Di Xue, Lifeng Chi, Lizhen Huang

**Affiliations:** State Key Laboratory of Bioinspired Interfacial Materials Science, Institute of Functional Nano and Soft Materials (FUNSOM), Soochow University, Suzhou, 215123 People’s Republic of China

**Keywords:** Transistor, Organic semiconductors, Negative photoconductance, Photonic synapse, Neuromorphic computing

## Abstract

The von Neumann architecture faces significant limitations, including low transmission efficiency and high energy consumption, when handling large-scale data and unstructured problems. Benefiting from the inherent merits of optical signals including high bandwidth, near-zero Joule heating, fast transmission speed, and immunity to electromagnetic interference, photonics provides a powerful pathway for high-speed neuromorphic computing. Together with the mechanical flexibility and largearea manufacturability of organic semiconductors, organic phototransistor (OPT)-based photonic synapses have therefore attracted extensive attention in recent years. This review provides a comprehensive overview of recent advances in OPT-based photonic synapses, covering operational principles, active materials, advances in bidirectional photoresponse process, as well as cutting-edge applications. Finally, the current challenges and opportunities in this field are highlighted. Distinct from previous reviews, this review emphasizes an in-depth exploration of bidirectional photoresponse mechanisms, a systematic dissection of material–structure–function correlations enabling integrated sensing-memory technology, and emerging.

**Graphical abstract:**

## Highlights

- The latest progress in neuromorphic artificial synapses based on organic phototransistors is reviewed from three aspects: functional semiconductor materials, operating behaviors, and frontier applications/advancements.
- The negative photoconductance behavior of novel phototransistors is discussed, along with their fascinating information-erasing capabilities demonstrated in organic photonic synapses.
- Frontier applications and advancements in neuromorphic vision driven by organic photonic synapses, such as human visual adaptation, polarization-sensitive detection, high-dimensional reservoir computing, and multimodal neuromorphic encryption, are demonstrated.

## Graphical abstract

**Figure:**
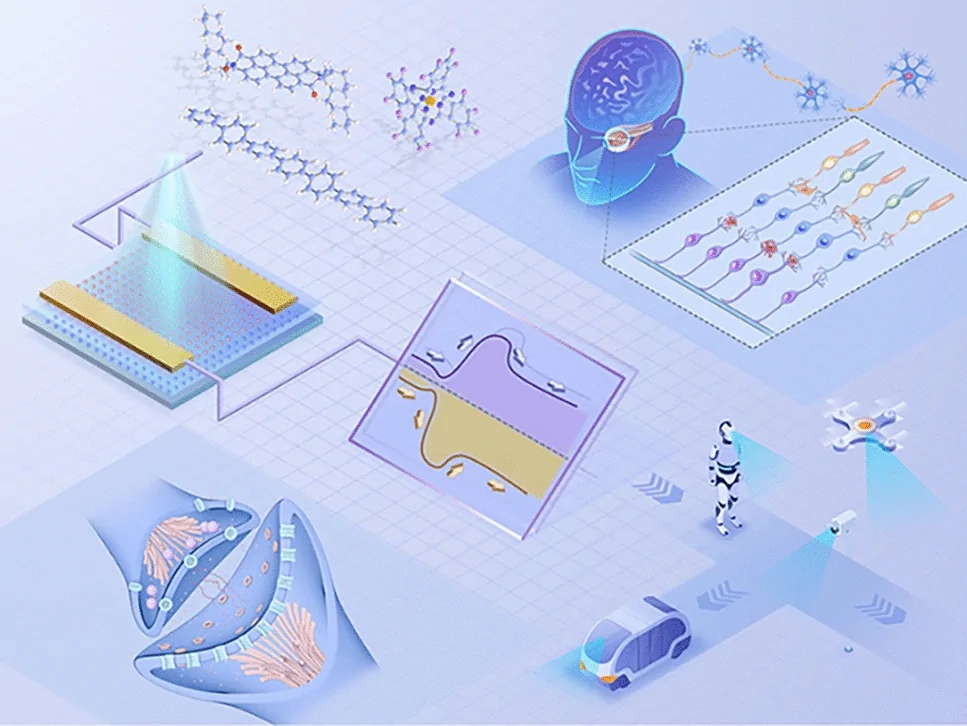

## Introduction

Since the emergence of the von Neumann architecture in the 1940s, its uncomplicated structure (composed of a central processing unit (CPU), memory, and input/output devices) and convenient operation flow (where the CPU fetches instructions from the memory and executes them sequentially) have significantly simplified the construction and management of early computers [1]. This has had a profound impact on the development of computing technology and software over the subsequent decades [2]. However, in the current era of booming big data, the separation of the CPU and memory in the von Neumann architecture means that data are transferred to the CPU or written back to the memory via the same channel. This situation has given rise to bottlenecks in the processing of vast amounts of data and unstructured problems. It not only restricts the data-processing speed, but also results in substantial energy consumption [3, 4]. To overcome this bottleneck, developing novel computing architectures and high-efficiency hardware is essential.

**Fig. 1:**
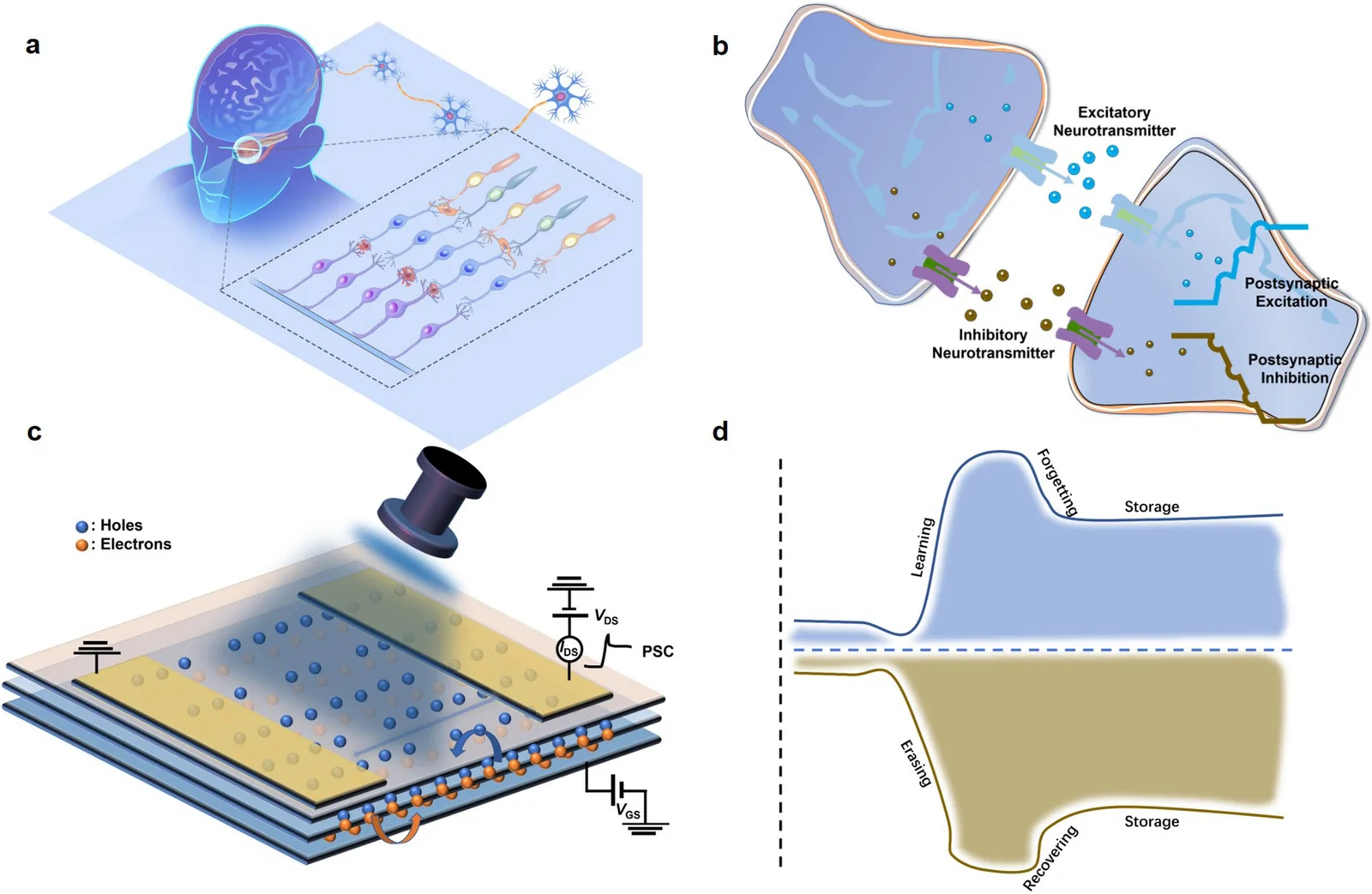
Principle of photonic synapses. **a** Humans receive, transmit, and possess visible information. **b** Biological synapse receiving excitatory/inhibitory neurotransmitters with postsynaptic excitation/inhibition. **c** Phototransistor responding to light. **d** Information learning and erasing process of a photonic synapse under positive or negative stimuli

Owing to its distinctive neural synapse network architecture, the human brain has outstanding parallel-processing capabilities, high efficiency, rapid response, and low power consumption [5–7]. Notably, the human brain can effortlessly handle massive and intricate unstructured problems, as well as probabilistic information, such as cognitive learning and image recognition (Fig. 1a, b). Consequently, taking inspiration from the human brain and fabricating artificial neural synapse devices that can imitate its data-processing paradigm and enable parallel processing and storage represents an ideal approach for addressing the current predicament [8].

Currently, artificial neural synapse devices that rely on electrical signal processing are mainly divided into two categories: two-terminal memristors [9] and three-terminal transistors. The latter include electrically stimulated floating-gate transistors (ESFGTs) [10], field-effect transistors (FETs) and electrochemical transistors (ECTs) [11], which have been shown to possess integrated capabilities for information processing and storage. Nevertheless, these devices are still constrained in terms of the speed of neuromorphic computing owing to their limited bandwidth density. In contrast, optical signals, characterized by high bandwidth, rapid transmission, and low energy consumption, are better suited to meet the demands of high-speed neuromorphic computing [12]. Furthermore, optically driven neuromorphic devices can mimic synaptic functions in the retinal neurons of the human eye [13], thereby replicating the biological mechanisms of light perception and information processing within visual systems. These photonic devices can detect external optical stimuli, generate images, and perform recognition and filtering operations to transmit optical information to downstream circuits, enabling image recognition, brain-inspired computation, and training processes. Notably, photonic synapses introduce the potential for contact-free writing strategies, which is conducive to the progress of wireless communication technologies. The phototransistors (Fig. 1c), integrated optical inputs as control signals [14] with three-terminated transistor configuration, can facilitate signal propagation and self-learning process, demonstrating promising applications in the artificial neural synaptic field [15, 16]. This article will concentrate on presenting recent advances in photonic synapse devices based on phototransistors, aiming to offer novel perspectives and inspiration for research and applications in relevant domains, thereby promoting the further development of this emerging and promising field.

Along with the development of photonic synapse devices, a wide range of novel photo-stimulated functional synaptic materials have been explored, including organic semiconductors [17–19], low-dimensional materials [20–22], perovskites [23], metal sulfides [24, 25] and metal oxides [26, 27]. Among them, organic semiconductor materials possess unique advantages, including low cost, abundant availability, solution processability, compatibility with low-temperature fabrication, tunable optoelectronic properties, excellent mechanical flexibility, and suitability for roll-to-roll large-area production [28, 29]. These properties position organic semiconductors as one of the promising candidates for developing large-area, integrated flexible photonic synaptic devices. Moreover, by hybridizing organic materials with other functional components—such as perovskites, quantum dots, nanowires, and carbon nanotubes—researchers can leverage their complementary electrical transport and photonic absorption properties to further enhance device performance through synergistic effects. This multi-material integration strategy not only mitigates the limitations of single-component systems, but also opens new paths for advancing the performance and functionality of photonic synaptic devices. Over the past decade, organic photonic synaptic transistors have garnered extensive attention and have achieved remarkable progress.

**Fig. 2:**
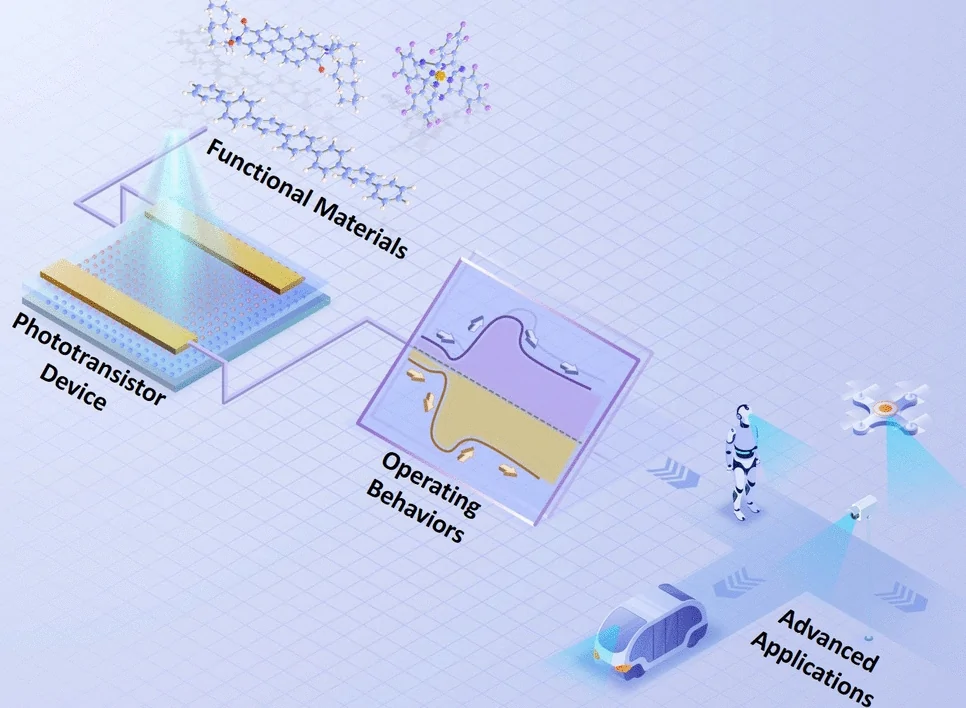
Organic photonic synapses and their materials, mechanisms, and applications

While several reviews have previously summarized this field, they have focused primarily on specific aspects. In 2021, Zhang et al*. *[15] provided a comprehensive overview of photonic synapses based on organic field-effect transistors, covering materials, devices, and applications. However, as the field was still in its infancy at the time, their discussion of potential applications has remained limited. Subsequently, Chen et al*.* [30] systematically reviewed the role of organic optoelectronic synaptic materials and devices in artificial visual sensing systems from different aspects, including functional layers (conductive channels, light-absorbing layers, luminescent layers), device structures (light-emitting diodes, phototransistors), and application domains (imaging memory, nociceptors, visual perception). This review focuses on organic phototransistor (OPT)-based synaptic devices, highlighting recent advances in active layer materials, bidirectional photoresponse behavior, and applications. We first elaborate on the key performance metrics of neuromorphic OPTs and their synaptic functional characteristics. Next, we summarize recent developments in organic functional materials and their hybrid combinations with other materials for photonic synaptic devices. Notably, we provide an in-depth analysis of the bidirectional photoresponse behavior—encompassing both positive photoconductance (PPC) and negative photoconductance (NPC) (Fig. 1d), which has received considerable attention recently. Furthermore, we highlight the latest breakthroughs in widely studied photonic synapses (Fig. 2) and explore potential avenues for functional expansion and integration in neuromorphic OPTs. Finally, we outline the current challenges and key research directions in the field, aiming to provide foundational insights and theoretical support for future advancements in multimodal computing, artificial vision systems, artificial intelligence, and the Internet of Everything.

## Key Performance Parameters of Photonic Synapses

To investigate and construct devices for intelligent systems with brain-like signal processing capabilities, neuromorphic devices require the ability to mimic the behavior and functions of synapses. In the nervous system of the human brain, synapses connect neurons and serve as important carriers for signal transmission and processing between neurons. A single synapse unit consists of a presynaptic membrane, a synaptic cleft, and a postsynaptic membrane [31, 32]. Synaptic activity can be briefly reflected in Fig. 1b and is understood as follows: When a presynaptic neuron receives a stimulus, it transmits an electrical signal to the presynaptic membrane. Upon receiving the signal, the presynaptic membrane releases neurotransmitters (such as ions, acetylcholine, dopamine, and amino acids) into the synaptic cleft through vesicles. When neurotransmitters move to the postsynaptic membrane, they are captured by specific receptors on the postsynaptic membrane, which then release or inhibit the release of electrical signals of corresponding intensities [33, 34]. Therefore, information in the nervous system can be carried by neurotransmitters and transmitted through the synapse units between neurons. Synapse units are the foundation for constructing complex neural networks of the human brain and realizing high-level neural network activities [35, 36]. For OPT devices, the source and drain electrodes usually correspond to the presynaptic membrane and the postsynaptic membrane, respectively (Fig. 1c). The optical signal can be regarded as the stimulus received by the previous synapse, and the channel current of channel conductivity between the source and drain electrodes corresponds to the neurotransmitters that transmit information. Table 1 shows a comparison of the synaptic properties of the biological synapse and phototransistor synapse devices. The key parameters that are typically evaluated include the excitatory and inhibitory postsynaptic current, synaptic plasticity and energy consumption. The following shows the detailed information of the three types of parameters.

**Table 1:** Summary of the synaptic properties of biological synapses and organic phototransistor-like synapses

Synapse type	Basic structure	Signal transmission	Synaptic activity	Synaptic Behaviors
Biological synapse	Presynaptic/postsynaptic membrane	Neurotransmitter	Postsynaptic excitation /inhibition	Paired-pulse facilitation (PPF) Paired-pulse depression (PPD) Short-term plasticity (STP) Long-Term Plasticity (LTP) Short-Term Plasticity to Long-Term Plasticity Transition (STP-to-LTP) Adjustment of Synaptic Weight (SW)
Artificial photonic synapse	Source-drain electrode/gate electrode	Conductive charge carrier	Excitatory/Inhibitory Postsynaptic Current (EPSC/IPSC)	

### Excitatory and Inhibitory Postsynaptic Currents

As mentioned above, neurotransmitters released into the synaptic cleft bind to receptors on the postsynaptic membrane. Specifically, when an action potential reaches the presynaptic membrane, voltage-gated calcium channels open, leading to an influx of Ca^2+^. This influx triggers the release of neurotransmitters from synaptic vesicles into the synaptic cleft. These neurotransmitters then bind to receptor proteins on the postsynaptic membrane, resulting in the opening of transmitter-gated ion channels. For instance, binding of an excitatory neurotransmitter causes depolarization of the postsynaptic cell, opening ion channels that permit the flow of positively charged ions (e.g., Na^+^ or K^+^), thereby generating an excitatory postsynaptic current (EPSC) [37, 38]. Conversely, when an inhibitory neurotransmitter binds to the receptor, the postsynaptic cell hyperpolarizes, and the membrane permeability to specific ions (such as Cl^−^) changes, producing a negative postsynaptic current known as an inhibitory postsynaptic current (IPSC) [39, 40]. The EPSC elevates the membrane potential above the resting potential, while the IPSC suppresses it below that level. In neuromorphic organic phototransistors (OPTs), an external optical stimulus can induce the generation or recombination of charge carriers in the conducive channel, leading to an increase or decrease in the channel current. After the light is removed, the altered channel current gradually decays over time. This decay is typically slow in photonic synapses, giving rise to memory-like behavior. These responses correspond to the EPSC or IPSC observed in photonic synaptic devices based on OPTs.

### Synaptic Plasticity

In biological systems, the degree of connection between neurons is defined as the synaptic weight (SW), which reflects the tightness of the connection between neurons and directly determines the magnitude of the postsynaptic current (PSC) [41]. The regulation of SW is the foundation of learning, cognition, and intelligence in the brain [42]. It depends on historical stimuli and can be precisely adjusted according to the activities of presynaptic and postsynaptic neurons, which is known as synaptic plasticity. Based on differences in duration, synaptic plasticity is generally classified into short-term plasticity (STP) [43, 44] and long-term plasticity (LTP) [45, 46]. In biological synapses, the PSC changes with varying stimulus duration. Usually, a stimulus pulse width of approximately several tens of milliseconds can effectively activate a synapse [47]. For photonic synapse devices based on OPTs, the PSC is not only related to the trapping and release behaviors of carriers, but also influenced by the gate voltage (*V*_G_) and drain voltage (*V*_D_). These voltage parameters can regulate the conductance state of the device, thereby affecting the magnitude of the PSC, which is crucial for mimicking the functions of biological synapses.

After a PSC is generated in a neural synapse under a single stimulus, applying another stimulus with a short time interval evokes a second PSC in the postsynaptic membrane. In this case, the PSC induced by the second stimulus will be larger or smaller than that induced by the first stimulus—a phenomenon termed paired-pulse facilitation (PPF) or paired-pulse depression (PPD) [48, 49]. In biological nervous systems, the PPF represents a critical manifestation of short-term plasticity (STP) and plays a crucial role in recognizing and decoding time-resolved information, such as visual signals [50, 51]. The magnitude of the PPF or PPD index is defined as follows:

1 $${\mathrm{PPF}} \, \text{or }\text{PPD }{\mathrm{index}}= \text{ } \frac{{\mathrm{A}}_{2}}{{\mathrm{A}}_{1}}$$

where A_1_​ is the peak value of the previous PSC, and A_2_​ is the peak value of the subsequent PSC. The dependence of the PPF or PPD index on the time interval follows the following double-exponential function:

2 $$\text{PPF or PPD }{\mathrm{index}}=C_{1}\mathrm{exp}\left(-\frac{\Delta t}{\gamma _{1}}\right)+C_{2}\mathrm{exp}\left(-\frac{\Delta t}{\gamma _{2}}\right)$$

where *C*_1_, *C*_2_ and *γ*_1_, *γ*_2_ represent the initial facilitation degree and the characteristic relaxation times of the fast and slow processes, respectively. For biological synapses, the value of *γ*_1_ is typically approximately one order of magnitude larger than that of *γ*_2_ [52]. On the basis of the PSC generated by a single light pulse in a photonic synapse, when a second light pulse is applied, the PSC induced by the second pulse will also be larger or smaller than that induced by the first light pulse. This phenomenon is called PPF or PPD in neuromorphic photonic synapses. In addition to the time interval, the PPF or PPD of photonic synapses is also affected by factors such as the *V*_G_, light intensity, and pulse wavelength.

The STP and LTP of biological and photonic synapses correspond to the short-term memory (STM) and long-term memory (LTM) proposed by Atkinson and Shiffrin in 1968, respectively [53, 54]. The synaptic connection strength in the STP state can only persist for milliseconds to minutes, whereas that in the LTP state can be maintained for an hour or even longer. Therefore, LTP is considered one of the fundamental mechanisms underlying the learning–memory transformation process. The memory level of the human brain is related to the intensity, duration, frequency, and number of learning experiences [55–57]. High-intensity, long-duration, high-frequency, and repeated learning can gradually strengthen the synaptic weight from weak (STP) to strong (LTP), leading to a transition from STP to LTP (STP-to-LTP). Similarly, photonic synapses can also increase the synaptic weight and achieve STP-to-LTP for memory functions by adjusting parameters such as the power density of the applied light stimulus, exposure duration, pulse frequency, and number of pulses.

### Energy Consumption

With the rapid development of artificial vision, there is an increasing demand for machine vision systems with real-time image recognition and intelligent analysis capabilities [58]. For photonic synapses, the memory function is more important than mobile portability [59, 60]. Therefore, in addition to considering the STP, LTP, and PPF mentioned above, the energy consumption of a single photonic synapse must be considered when designing photonic synapses.

The energy consumption of a single synaptic event simulated by traditional CMOS circuits is approximately 900 pJ [61], whereas the human brain only consumes 1–100 fJ per synaptic event during large-scale efficient parallel information processing [62]. One of the critical significances of developing neuromorphic computing lies in its low energy consumption for computation. Low energy consumption represents one of the most challenging performance metrics in the research of neural synaptic devices. The most common formula for calculating the energy consumption per single synaptic event in synaptic devices is:

3 $$E={\int }_{t_{0}}^{t_{1}}V\cdot I\left(t\right)\cdot \mathrm{d}t$$

where *t*_0_ and *t*_1_ are the start and end times of light application, *V* is the voltage applied to the device, and *I* is the output current of the device. This method accounts for the energy consumption of the photonic synapse device during its electrical response to light stimulation, indicating that for a given device voltage, the smaller the photocurrent or the shorter the light stimulation time is, the lower the energy consumption of the device [4, 30, 62].

Overall, optimization of synaptic performance in OPT devices can be guided by several general principles:

- 1.Enhancing excitatory/inhibitory postsynaptic currents relies on improving photocarrier generation and transport through band-gap engineering, interfacial charge transfer design and dielectric modification.
- 2.Synaptic plasticity can be tuned by tailoring trap density, trap energy levels and carrier retention pathways, enabling controllable transition from short-term to long-term memory.
- 3.Reducing energy consumption requires minimizing the operational voltage and photocurrent by engineering charge-storage efficiency and leveraging optical-gating effects.

These strategies not only provide a unified guideline to balance responsivity, memory retention and energy efficiency for neuromorphic vision applications, but also establishes the basis for materials and device structure designs discussed in the following sections.

## Active Materials in Organic Phototransistors

For photosensitive materials in synaptic phototransistor, the generation of photocurrent and its dynamic process are critical for the synaptic applications [63, 64]. The magnitude of the photocurrent depends on the number of photogenerated charge carriers, which is closely related to the bandgap of the semiconductor material and the energy of the incident light [65, 66]. According to the classical Einstein photoelectric effect equation, the energy of the incident light is inversely proportional to its wavelength. Therefore, when designing OPT-based photonic synapses, both the wavelength of the light pulse and the band gap of the organic semiconductor material are necessary to consider. An appropriate band gap is the basis for generating photogenerated carriers and determines effective charge transfer. In addition, as transistor devices, efficient charge transport is required to enable field effect operation and high photoresponsivity.

As mentioned previously, one of the advantages of organic materials is the tunability of their band gaps. Specific functions can be imparted to organic semiconductor materials through molecular design and chemical modification. For example, in terms of conductivity, the structure of organic semiconductor molecules features a large π-conjugated system, which contains many delocalized electrons. These electrons can move freely within the conjugated structure and can also jump to adjacent molecules through intermolecular interactions such as van der Waals forces. In organic semiconductor molecules, the benzene ring is the most typical π-conjugated system. When photonic synapses are designed, porphyrins, phthalocyanines, acrylic-based derivatives, and oligothiophene dendrimers are good alternative building blocks for designing photonic synapse molecules [29]. In addition, considering the preparation process, for organic semiconductor molecules with relatively low molecular weights, thermal evaporation is often used to prepare semiconductor thin films, whereas for those with relatively high molecular weights, the solution method is a better choice, where researchers often add alkyl chains during molecular design [67].

**Fig. 3:**
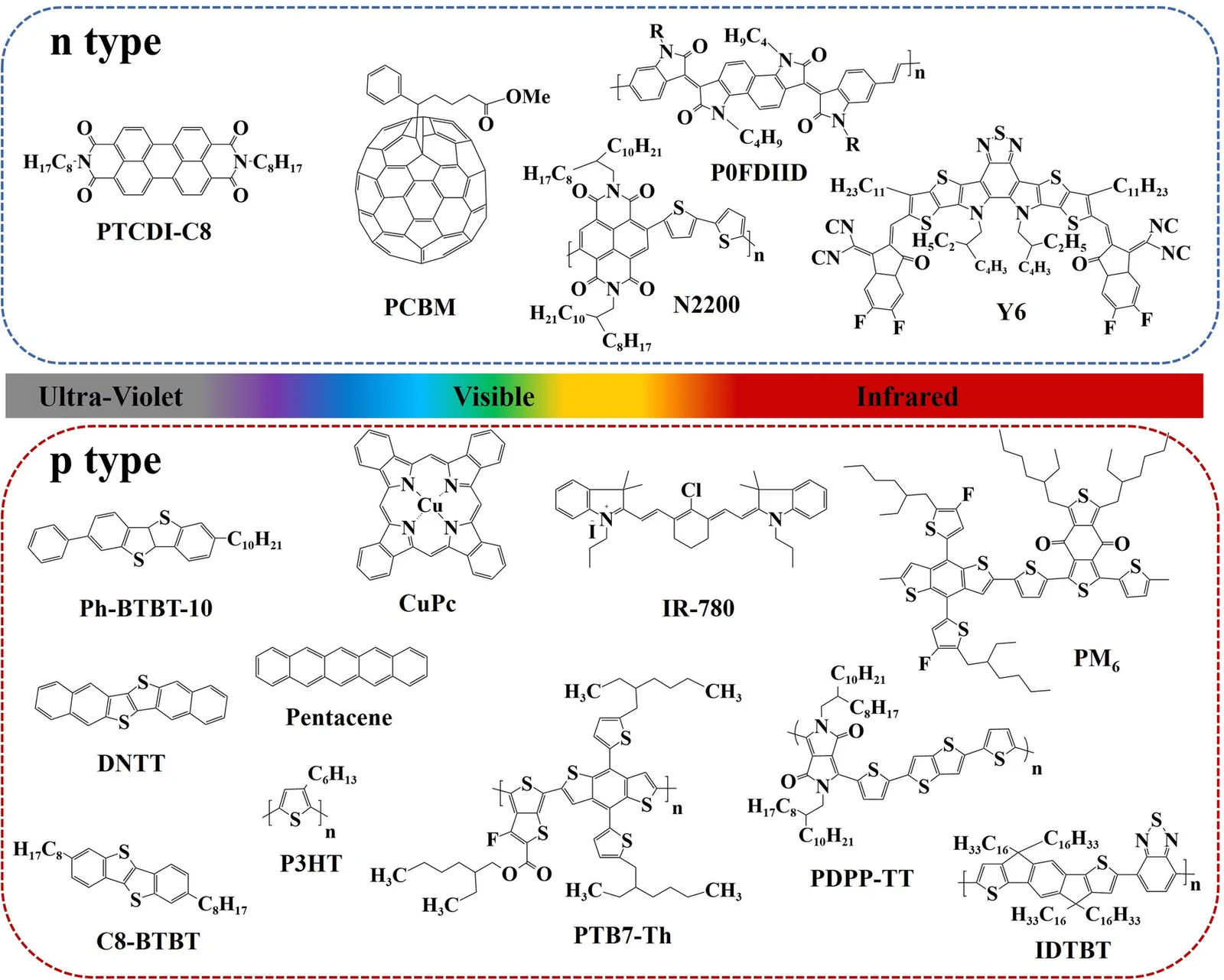
Organic semiconductor materials applied in OPT-based photonic synapses in recent years

In recent years, the functional organic semiconductor materials applied in OPTs can be broadly classified into three categories: single-component semiconductor materials, bulk heterojunctions (BHJs) that mix n-type and p-type semiconductors, and planar heterojunctions (or van der Waals heterojunctions). Figure 3 summarizes various active-layer organic semiconductor materials used in OPT-based photonic synapses reported in recent years, including channel active layer and photosensitive materials. For single-component semiconductors, strong intermolecular forces create low-energy potential dissociation sites for charge separation, which reduce the barrier for excitons to split into charge pairs [68]. The efficiency of charge generation mainly depends on the migration of excitons to these sites. These materials have simpler preparation processes and lower costs. However, single-component semiconductors perform poorly in terms of effective exciton dissociation and usually can only exhibit unidirectional PPC responses because of their homogeneous composition. Moreover, single-component semiconductor materials rarely exhibit both high electrical conductivity and excellent photosensitive properties. BHJs contain donor–acceptor (D-A) molecules, where photoinduced charge separation occurs at the D-A interface. Electrons are retained in the lowest unoccupied molecular orbital (LUMO) of the acceptor, and holes remain in the highest occupied molecular orbital (HOMO) of the donor. The efficiency of charge separation depends on the degree of mixing between the acceptor and donor: a high degree of mixing shortens the diffusion length, increasing the photogeneration rate, whereas a certain degree of demixing ensures effective charge collection [69]. Notably, the nonvolatile memory behavior of photonic synapses is fundamentally governed by carrier trapping at heterojunction interface traps. Planar heterojunction structures provide a platform for electron transport, and by designing the bandgap distribution of different materials, the retention time of carriers in the heterojunction can be extended to mimic synaptic storage functions [70, 71]. In addition to pure organic‒organic heterojunctions, researchers have explored other materials, such as inorganic materials, perovskites, quantum dots, nanowires, and carbon nanotubes, to construct organic‒inorganic hybrid structures with superior electrical conductivity and more comprehensive photosensitive properties. By constructing heterojunctions between organic semiconductors and these materials, various synaptic functions can be mimicked, enabling applications in artificial vision. This section introduces recent advancements in OPT-based photonic synapses for the abovementioned material systems.

### Organic Semiconductor Materials

The synaptic properties of most photonic transistors based on OPTs are determined by the appropriate degree of charge trapping between organic semiconductor materials or between organic semiconductors and dielectric layers [72, 73]. Therefore, in terms of device structure, most researchers have chosen to adopt the bottom-gate top-contact configuration. This is not only because this structure is relatively easy to construct, but also because the Si/SiO_2_ substrate provides a natural gate and dielectric layer, which facilitates the realization of synaptic properties [4, 74]. The careful design of heterojunctions can enable reversible charge transport and the storage of electrons over a period of time. Therefore, by combining high-photosensitive materials with high-charge-transport materials, the synaptic performance of fabricated OPT devices often provides more functionality and a wider spectral response range than single-material devices do.

**Fig. 4:**
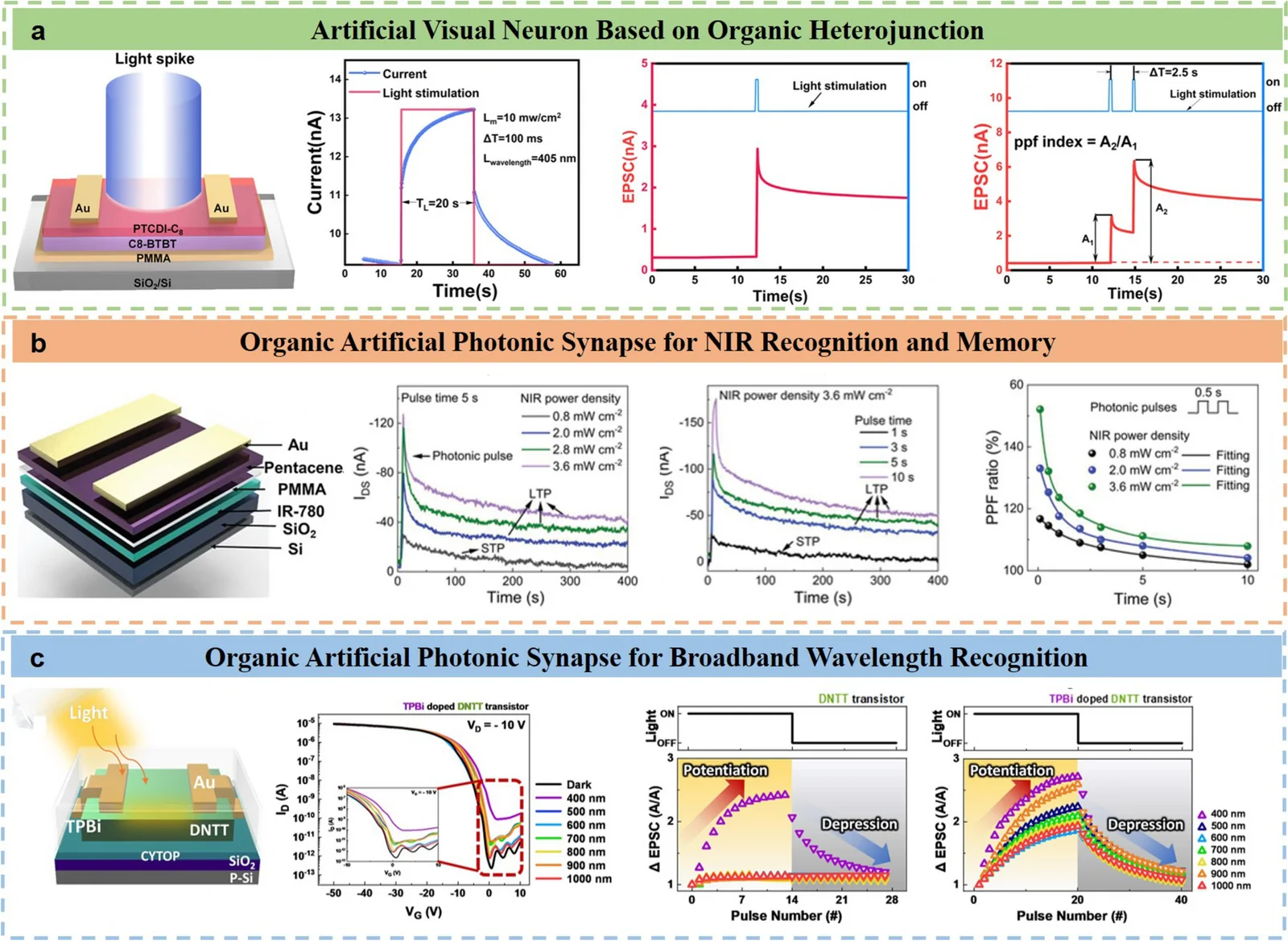
A summary of OPT-based photonic synapses that respond to light with different wavelengths. **a** Artificial visual neuron based on organic heterojunction. Reproduced with permission [75] Copyright (2024), American Chemical Society. **b** Organic artificial photonic synapse for NIR recognition and memory. Reproduced with permission [76] Copyright (2021), Wiley–VCH GmbH. **c** Organic artificial photonic synapse for broadband wavelength recognition. Reproduced with permission [77] Copyright (2024), American Chemical Society

The flicker frequency of incident light is a critical determinant in biology [78]. However, exploring methods to simulate external light stimuli of different frequencies and develop artificial retinal neurons with responsive behaviors remains an unresolved issue. In 2024, Wang et al*.* [75] reported an artificial visual neuron (AVN) based on a PTCDI-C_8_/C8-BTBT heterojunction, which exhibited excellent synaptic functions under 405 nm pulsed light stimulation (Fig. 4a). The vapor-deposited PTCDI-C_8_/C8-BTBT heterojunction serves as a photosensor, where the PTCDI-C_8_ film induces a charge-trapping effect to receive light signals and convert them into electrical signals output through the light source. The processes of photogenerated charge trapping and release at the interface mimic synaptic-like behaviors. The EPSC and EPSCs triggered by two consecutive optical spikes represent typical synaptic memory, where the EPSC induced by the second spike is larger than that induced by the first, resembling the PPF. On this basis, by constructing an external mobile platform to adjust the movement speed of the light source, the device accurately replicates different frequencies of light stimulation from the external environment, generating EPSCs that resemble biological visual-perception curves and reproduce the persistent behaviors observed in biological visual systems. Finally, through circuit integration, the potential application of the PTCDI-C_8_/C8-BTBT AVN in motion detection is explored to achieve obstacle avoidance functions in mobile robotic vehicles.

High-sensitivity near-infrared (NIR) optical recognition and analysis play critical roles in defense and civilian applications, such as night surveillance, information encryption, data security, and medical imaging [79–82]. Mu et al*. *[76] adopted the organic material IR-780 iodide as the charge-trapping layer for artificial synapses via OPTs (Fig. 4b). This photonic synapse integrates both NIR recognition and memory functions. NIR incident light signals with different intensities and durations can be converted into volatile STP and nonvolatile LTP synaptic signals, resulting in excellent synaptic plasticity. Moreover, by integrating an IR-780 iodide-based artificial synapse with a leaky integrate-and-fire (LIF) neuron via Ag/SiO_2_/Ag electrodes, an artificial sensory neuron system that encodes NIR light information into electrical pulses was constructed. Additional NIR radiation stimulation of artificial synapses can significantly increase the spike rate. On this basis, a spiking neural network (SNN) for handwritten digit classification was further constructed.

In addition to the heterojunction OPT-based photonic synapses in the above wavelength ranges, Kang et al*.* [77] proposed a small-molecule OPT photonic synapse based on a TPBi-doped DNNT/CYTOP heterostructure for broadband wavelength recognition (Fig. 4c). Under normal conditions, the DNTT transistor shows no obvious changes across the entire wavelength spectrum except in the 400 nm range, which is consistent with the absorption region of DNTT. However, the TPBi-doped DNTT phototransistor exhibits an enhanced off-current in its transfer curves and shows photoresponse behavior across the entire wavelength range of 400–1000 nm. Conventional DNTT transistors display consistent reading current levels under light illumination in the 500–1000 nm wavelength range. In contrast, the TPBi-doped DNTT phototransistors show an increase in the reading current under light of all wavelengths. Based on these findings, an artificial neural network (ANN) was constructed and used for facial recognition simulation by leveraging synaptic behaviors. Artificial synaptic behaviors were successfully mimicked by distinguishing the off-current levels, achieving a recognition accuracy exceeding 70% across all wavelength ranges.

### Organic–Inorganic Hybrid Structure

By jointly applying organic semiconductors with other materials in OPT-based photonic synapses, various optical synaptic devices based on inorganic perovskites, quantum dots, and nanostructures have shown great significance in optical information processing. Owing to their excellent optoelectronic properties, such as high stability, high luminescence performance, and excellent light absorption performance, perovskite quantum dots have attracted extensive attention [83]. Owing to their unique wide-range optical response, excellent electrical properties, strong stability, and, more importantly, easy formation of large-scale arrays, nanowires are expected to have promising applications in artificial synaptic devices [84–87]. Kim et al*.*  [88] studied the synthesis of semiconductor nanowires with functional hybrid nanostructures with tunable optical properties by controlling the formation of spatially controlled self-assembled nanowires on the basis of poly(P3HT)-b-poly(2-vinylpyridine) rod‒coil amphiphiles and CdS quantum dots (QDs). In addition, Ren et al*.* [89] demonstrated hybrid anisotropic nanowires of P3HT and CdS quantum dots through chemical grafting and ligand exchange. With improvements in purity, quality, and scalable manufacturing techniques, carbon nanotubes have become an ideal semiconductor material for transistor manufacturing and large-scale neuromorphic systems [90–93]. In the following, experimental cases in which quantum dots, nanostructures, etc., have been applied to OPT-based photonic synapses in recent years are introduced. This section will focus on hybrid systems composed of organic semiconductors and various functional materials, which may incorporate bulk heterojunction or planar heterojunction architectures.

#### Organic Semiconductor/Perovskite

**Fig. 5:**
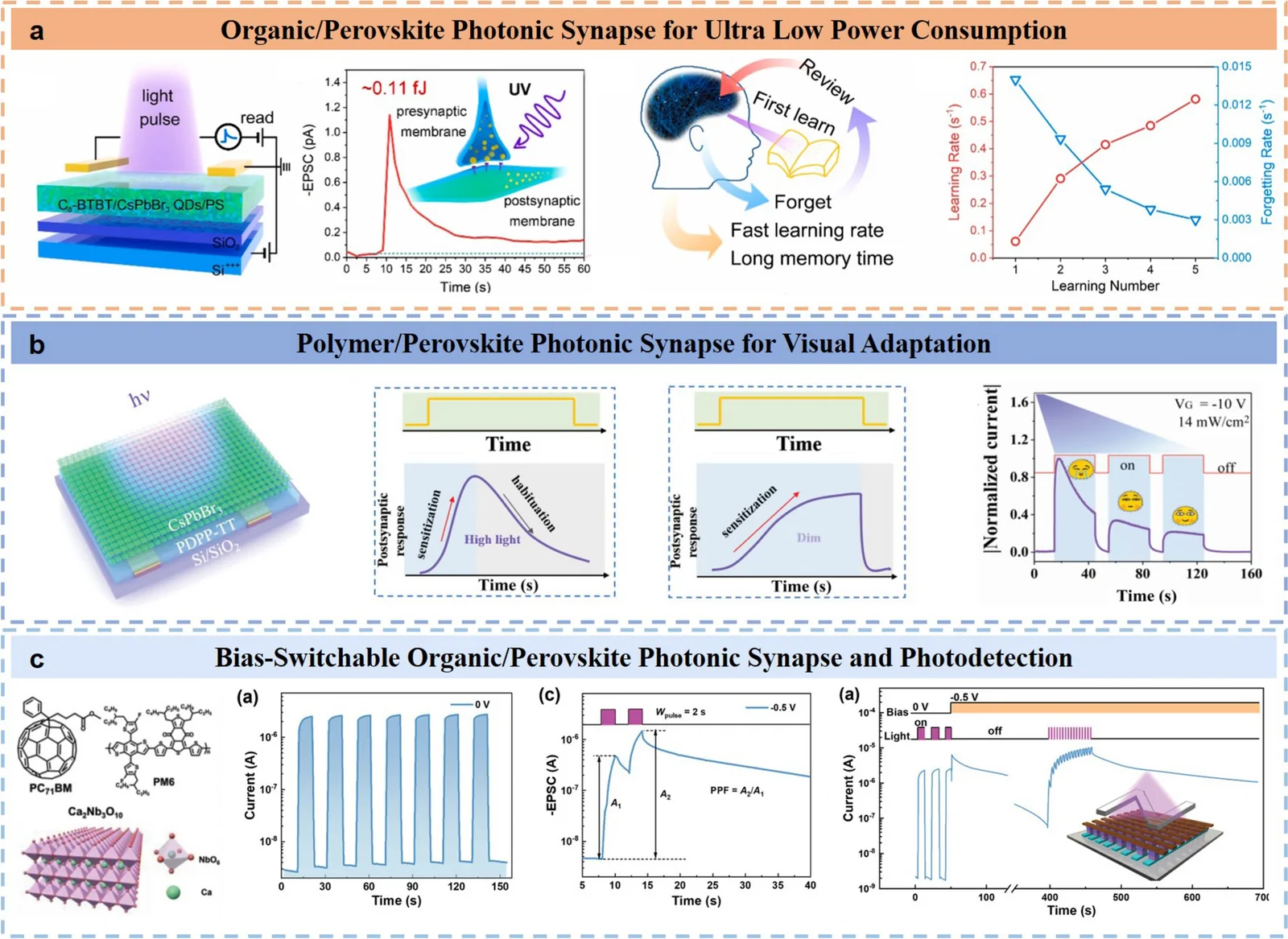
Photonic synapses based on the combination of organic semiconductors and perovskite materials. **a** Organic/perovskite photonic synapse for ultra-low power consumption. Reproduced with permission [99] Copyright (2021), Elsevier Ltd. **b** Polymer/perovskite photonic synapse for visual adaptation. Reproduced with permission [100] Copyright (2022), Wiley–VCH GmbH. **c** Bias-switchable organic/perovskite photonic synapse and photodetection. Reproduced with permission [101] Copyright (2024), Wiley–VCH GmbH

As candidate materials for photonic synapses, perovskite-based composite materials offer advantages of low cost, processability, and excellent optoelectronic properties, including high photoluminescence quantum yield, narrow-band emission, high dielectric constant, and tunable optical bandgap [94–98]. Shi et al. [99] proposed a multifunctional synaptic transistor based on a ternary organic semiconductor (OSC)-polymer-inorganic perovskite quantum dot (IPQD) photosensitive layer (Fig. 5a). A novel ternary film was constructed using C8-BTBT with high crystallinity for charge transport and high UV responsiveness, IPQDs with enhanced UV absorption and charge transfer functions, and PS polymers with enhanced charge lifetime functions. Owing to the dual light-absorption function of OSCs and IPQDs, this synaptic device exhibits a minimum power consumption of *≈* 0.11 fJ per spike at a low operating voltage of − 0.01 V, along with remarkable optical synaptic-like behaviors featuring adjustable STP and LTP. The addition of polymers improved the device stability and printability of the ternary solution. The synaptic device was further characterized to demonstrate learning-forgetting behaviors, Morse code information processing, and pattern recognition capabilities tested on the MNIST dataset.

Kuang et al*.* [100] proposed an interface defect tuning strategy to fabricate a novel dual-function phototransistor (Fig. 5b). The self-assembly tuning of perovskite quantum dots (PQDs) can ensure good interface contact with the semiconductor layer, thereby improving the injection efficiency of photogenerated carriers. The modulated defect states at the polymer‒perovskite interface promote the trapping and release of photogenerated charges, endowing the transistor with ideal optical synaptic characteristics. The fabricated phototransistor perfectly mimics human visual nerve behaviors, including PPF, STP, and LTP. Moreover, these devices simulate the transition from STP-to-LTP through a strength training process. The phototransistor has high photosensitive performance and a fast response speed (< 35 ms, which is far lower than the response speed of the human eye to incident light (225 ms)). More importantly, the phototransistor exhibits obvious visual adaptation behavior under dynamic wide-range illumination and achieves unique desensitization similar to the self-protection behavior of the human visual system. Specifically, the adaptation time scales of the device under weak and strong light conditions can precisely match those in biological systems (< 2 min).

Integrating high-sensitivity photodetection with photonic synaptic characteristics in a single device can significantly enhance the overall integration and efficiency of artificial visual systems (AVSs) [102, 103]. However, owing to the fundamentally different working mechanisms of photodetectors and photonic synapses, this integration poses significant challenges. Zhao et al*.* [101] developed a bias-switchable photodetection and photonic synaptic device using a 2D perovskite oxide/organic heterojunction (Fig. 5c). This unique structure allows modulation of carrier dynamics under different bias conditions, enabling the device to function as a photodetector without bias and as a photonic synapse with bias. At zero bias, the device achieves high responsivity (≈ 0.36 A W^−1^ at 320 nm) and a fast response speed (0.57 s). Under a − 0.5 V bias, it exhibits persistent photoconductivity (PPC), leading to neuromorphic synaptic behaviors with a PPF index exceeding 300%. Additionally, an 8 × 8 sensor array demonstrates image sensing and storage capabilities, showing in situ enhanced imaging when switching the bias from 0 to − 0.5 V and achieving image storage for over 200 s. AVS was further explored for image processing and recognition by constructing a 28 × 28 device array integrated with an ANN. The adjustable synaptic weights under different reverse biases allow optimized analog recognition, achieving 92% accuracy after 160 training epochs.

#### Organic Semiconductor/Quantum Dots

**Fig. 6:**
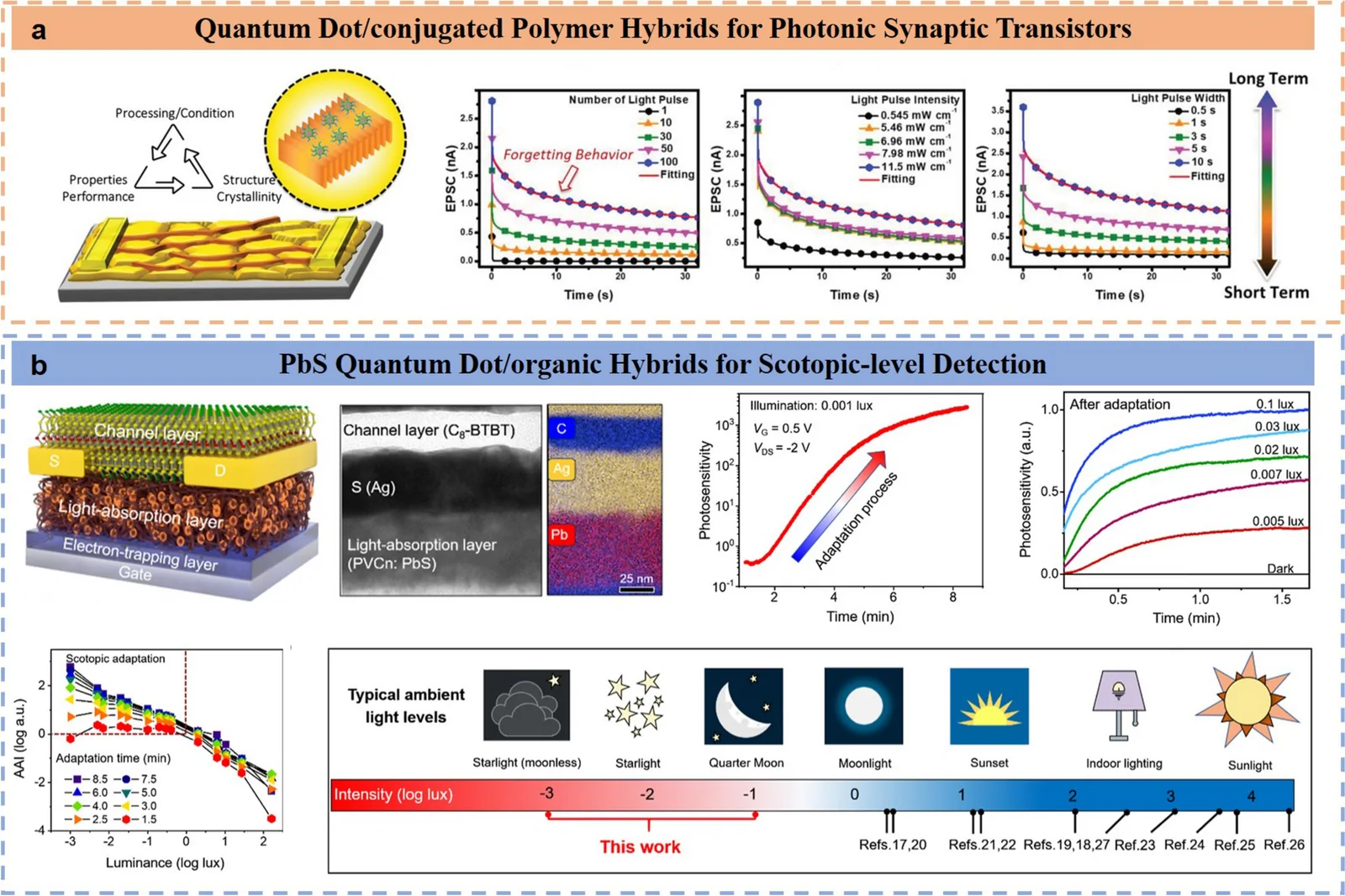
Photonic synapses based on the combination of organic semiconductors and quantum dot materials. **a** Quantum dot/conjugated polymer hybrids for photonic synaptic transistors. Reproduced with permission [104] Copyright (2021), Wiley–VCH GmbH. **b** PbS quantum dot/organic hybrids for scotopic-level detection. Reproduced with permission [105] Copyright (2024), American Chemical Society

The charge dissociation and optical properties of excitons can provide a deeper understanding of the relationship between nanostructures and their charge transport properties. The study of the effects of morphological and structural interactions, including molecular packing, interfacial charge separation, and transport between quantum dots and semiconductor polymers, as well as the use of solution pretreatment for the composite perovskite-P3HT aggregate system in photonic synaptic transistors, may be beneficial for low-voltage-driven and low-energy-consuming devices because of better charge dissociation within the QD distribution on the nanofibers [96, 97, 106]. Influenced by the uniform axial distribution of quantum dots and the formation of poly(3-hexylthiophene) (P3HT) nanorods and coaggregates, the photonic synaptic device based on perovskite (CsPbBr_3_) QD/P3HT composite nanofiber films (CNFs) reported by Ercan et al*.* [104] exhibits excellent performance (Fig. 6a). Charge dissociation and photonic synaptic performance were improved through solution processing and treatment, such as the addition of edge solvents, sonication, and UV treatment. The photonic synaptic transistor with CNFs can perform basic functions, including STP and LTP, to simulate sensing, computing, and memory functions. Notably, the synaptic device using CNFs has an ultralow energy consumption of 0.18 fJ and can operate at zero gate voltage. This study provides a new perspective for the fabrication of artificial synapses with one-dimensional self-assembled nanostructures.

Owing to biological feed-forward adaptation, human vision excels at perceiving low-light conditions at night [107, 108]. There has been a long-standing enthusiasm for replicating this ability in bionic vision via solid-state devices. However, mimicking dark-adaptation vision requires a combination of efficient photoexcitation and dynamic carrier modulation, which poses significant challenges [109, 110]. Inspired by human dark-adaptation vision, Luo et al*.* [105] reported an organic scotopic adaptation transistor (OSAT) that can simultaneously achieve efficient generation of free photocarriers and adaptive carrier accumulation within a single device by coupling a light-absorbing layer (PVCn: PbS quantum dots) and an electron-trapping layer at the bottom of the semiconductor channel (Fig. 6b). This innovation endows the transistor with enhanced sensitivity after adaptation, enabling it to detect scotopic-level illumination (0.001 l ×) with excellent photosensitivity of up to 10^3^ at a low voltage below 2 V. On the basis of these adaptation characteristics, various scotopic vision functions observed in human vision, including adaptation-time-related visual threshold enhancement and light-intensity-related adaptation facilitation, were successfully realized in the OSAT. Moreover, they constructed an artificial visual system consisting of a 10 × 10 OSAT active array with environmental adaptation capabilities. Over time, this system has exhibited high-contrast imaging capabilities in low-light environments, similar to the characteristics of human scotopic vision. In general, they successfully replicated various scotopic vision functions, including time-related visual threshold enhancement, light-intensity-related adaptation indices, and enhanced imaging contrast for low-light imaging at night.

#### Organic Semiconductor/Other Functional Materials

In addition to quantum dots, inorganic metal oxide or carbon-based materials are frequently employed to construct organic/inorganic hybrid structures and demonstrate diverse functionalities.

**Fig. 7:**
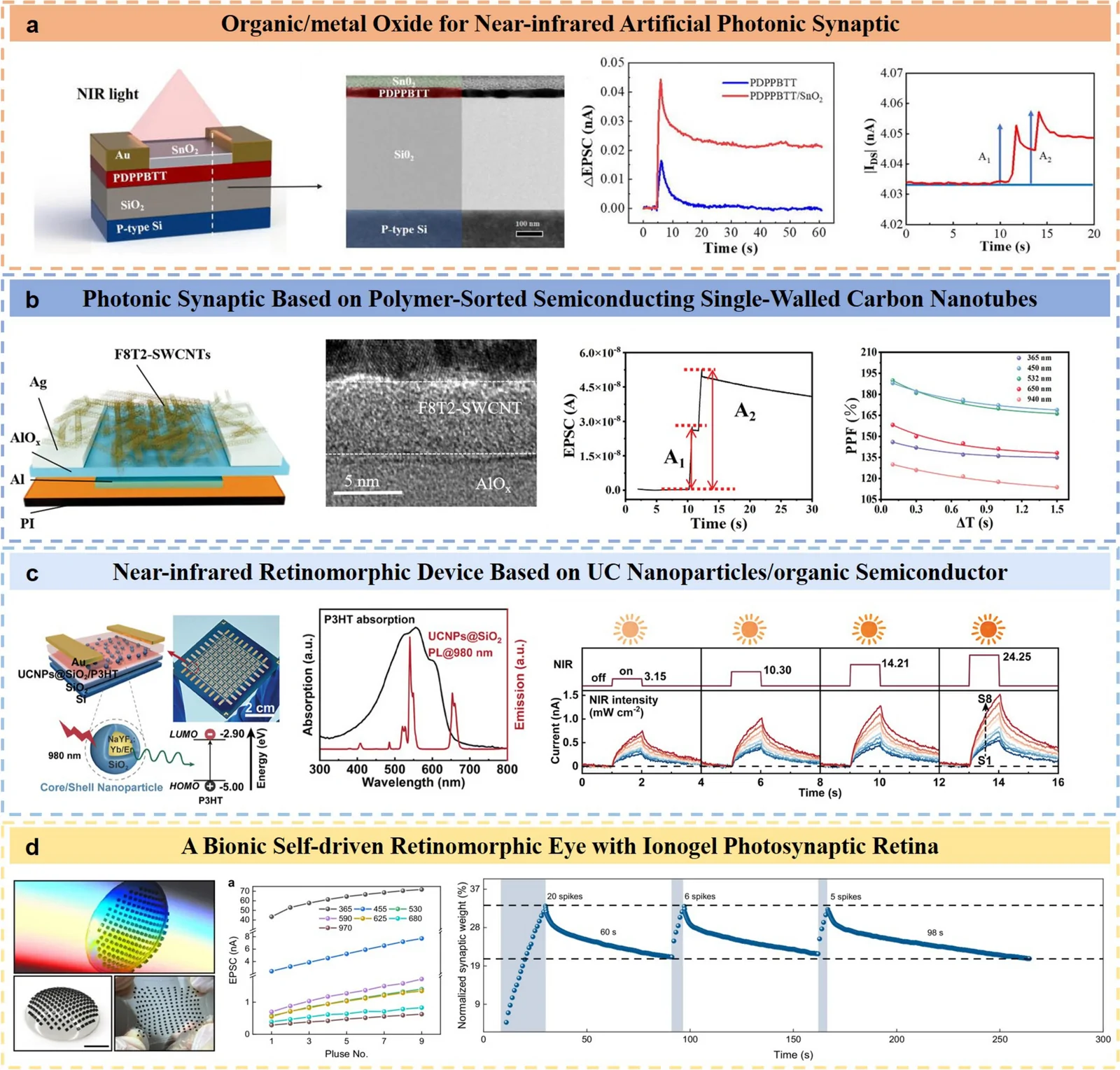
Photonic synapses based on the combination of organic semiconductors and other functional materials. **a** Organic/metal oxide for near-infrared artificial photonic synaptic. Reproduced with permission [111] Copyright (2022), AIP Publishing. **b** Photonic synaptic based on polymer-sorted semiconducting single-walled carbon nanotubes. Reproduced with permission [112] Copyright (2024), Wiley–VCH GmbH. **c** Near-infrared retinomorphic device based on UC nanoparticles/organic semiconductor. Reproduced with permission [113] Copyright (2024), Wiley–VCH GmbH. **d** A bionic self-driven retinomorphic eye with ionogel photosynaptic retina. Reproduced with permission [114] Copyright (2024), Spring Nature

Sha et al. [111] designed an NIR photonic synapse based on an organic/inorganic heterojunction phototransistor, which consists of a simple donor (p-type polymer semiconductor PDPPBTT)/acceptor (inorganic SnO_2_) (D/A) heterostructure (Fig. 7a). By combining acceptor materials such as polar-polymers-poly(methyl methacrylate) (PMMA), polylactic acid (PLA), ZnO nanocrystals, and fullerene derivatives with donor materials, the efficiency of exciton dissociation can be enhanced because of electron-trapping moieties in the polymer or energy offsets between the donor and acceptor. Therefore, the PDPPBTT/SnO_2_ heterojunction structure enables the customization of charge generation, separation, and transport in OPTs to achieve superior NIR light-response characteristics, providing a critical prerequisite for high-performance NIR photonic synapses. Under NIR illumination, the charge-trapping effect at the PDPPBTT/SnO_2_ interface triggers fundamental synaptic behavior. This study demonstrates the basic functions of light-stimulated artificial synapses, such as EPSCs and PPFs, and reveals a simple device structure and fabrication process to realize efficient NIR photonic synapses for high-performance visual systems.

Conjugated polymers can spontaneously wrap around semiconducting single-walled carbon nanotubes (sc-SWCNTs) and effectively sort sc-SWCNTs, with polymer encapsulation preventing charge dissipation in sc-SWCNT devices. In 2024, Sui et al. [112] adopted a scalable preparation method to fabricate a light-programmable multifunctional optoelectronic synaptic transistor array using the photosensitive polymer poly[(9,9-dioctylfluorenyl-2,7-diyl)co(dithiophene)] (F8T2)-sorted sc-SWCNT as the active layer (Fig. 7b). This optoelectronic device achieves excellent broadband photonic synaptic characteristics (from 365 to 940 nm), multilevel storage (200 conductance states), and facile synaptic state switching. Based on these broadband optoelectronic synaptic properties, a new SNN algorithm was developed to perform recognition tasks on the Caltech 101 dataset, completing multi-featured image recognition faster (only ≈70 epochs) and more accurately (up to 97.92%).

Intelligent perceptual reservoir computing (RC) systems based on physical reservoirs have recently garnered attention because of their low computational resource requirements [115–119]. However, such systems remain limited in infrared (IR) machine vision, including material and physical reservoir expressiveness [120–124]. Inspired by biological visual-perception systems, Leng et al. [113] proposed an NIR retinal device capable of simultaneously sensing and encoding narrow NIR spectral information (≈ 980 nm) (Fig. 7c). The device features a core‒shell up conversion nanoparticle/P3HT nanocomposite channel, which absorbs and converts NIR light into high-energy photons to excite more photocarriers in P3HT. Photon‒electron coupling dynamics under the synergistic effects of photovoltaic and photogating mechanisms endow the RC system with nonlinearity and high dimensionality under narrow-band NIR irradiation. The device also exhibited multilevel data storage capability (≥ 8 levels), excellent stability (≥ 2000s), and durability (≥ 100 cycles). It accurately recognizes static and dynamic handwritten digit images in the NIR range, achieving recognition accuracies of 91.13% and 90.07%, respectively. Consequently, the device can solve complex computations such as second-order nonlinear dynamic equations with minimal error (normalized mean squared error of 1.06 × 10⁻^3^ during prediction).

Bioinspired eyes should possess characteristics such as autonomous operation, repairability, and adaptability to arbitrary geometries. These eyes can achieve wide-field detection and efficient visual signal processing without external energy while also enabling retinal transplantation to restore vision by replacing dysfunctional photoreceptors with healthy photoreceptors [125]. Although various artificial eyes have been constructed using hemispherical silicon, perovskite, and heterostructure photoreceptors, creating a zero-power retinal system with portable conformal features remains challenging [126, 127]. By integrating neuromorphic principles with retinal and ionic elastomer engineering, Luo et al. [114] demonstrated a self-driven hemispherical retinal eye, where the elastic retina is fabricated from an ionogel heterojunction serving as the photoreceptor (Fig. 7d). A photosensitive heterojunction was developed by selectively doping ionogel pillars with polypyrrole nanoparticles (PPy-NPs). The pillar array can be directly implanted onto the surface of a transparent ionogel hemisphere facing incident light, forming a retina-like columnar forest with both photoelectric conversion capability and neuroelectric plasticity. The self-powered characteristic is primarily provided by photothermoelectrically induced ion drift within the ionogel, differing from previous self-powered optical synapses induced by the photovoltaic effect, thus showing great potential in energy-efficient autonomous sensing technologies. Additionally, all soft components endow the retina with excellent conformal and stretchable capabilities, allowing it to adhere to any object with complex geometries. The system exhibits broadband light detection (365–970 nm), a wide field of view (180°), and photonic synaptic behavior (PPF, 153%), making it suitable for bioinspired visual learning. Retinal photoreceptors are portable and adaptable to any complex surface, enabling visual restoration for dynamic optical imaging and motion tracking. This approach provides a simple and effective method to construct zero-power photodetectors, particularly portable organ-like conformal retinal photoreceptors.

**Fig. 8:**
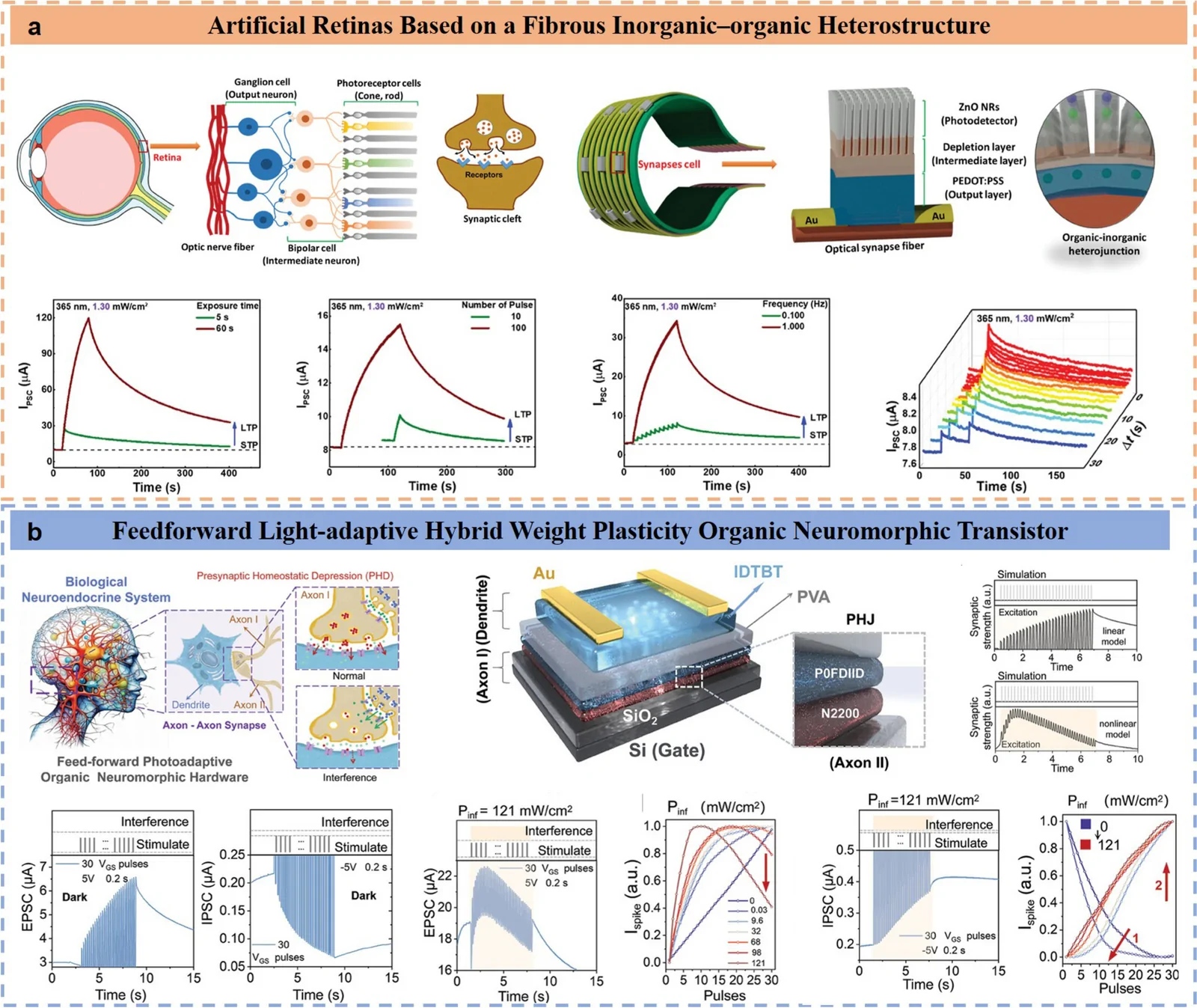
Examples of biostructure-inspired photonic synapses. **a** Artificial retinas based on a fibrous inorganic–organic heterostructure. Reproduced with permission [128] Copyright (2023), Wiley–VCH GmbH. **b** Feedforward light-adaptive hybrid weight plasticity organic neuromorphic transistor. Reproduced with permission [129] Copyright (2024), Wiley–VCH GmbH

The development of flexible fiber-based artificial synaptic sensing devices with multiple basic synaptic functions and stable mechanical/electrical properties represents a necessary first step toward wearable neuromorphic perception [130–132]. Considering that fiber structures play crucial roles in major biological organs—such as optic nerve fibers in the eye, nerve fibers in the brain’s high-density neural network, and peripheral nerve fibers in the limbs—the development of retina-inspired artificial synapses on fiber platforms is highly important, as it expands the potential applications of wearable fiber-structured neuromorphic sensory devices [133–136]. Lee et al. [128] proposed the design and fabrication of a fiber photonic artificial synapse (FPAS) (Fig. 8a). By constructing a vertical heterojunction structure of ZnO nanorods (NRs) and poly(3,4-ethylenedioxythiophene) polystyrene sulfonate (PEDOT:PSS) on polyurethane (PU) fibers, FPAS can mimic the structure of the human retina and optic nerve, as well as the operational principles and synaptic functions of the optic nerve. The FPAS can be easily integrated into fabrics or coiled around tubes and remains functional under mechanical bending stress and after cyclic bending without significant degradation of synaptic properties. This photonic synaptic device also exhibits distinct basic optical synaptic functions through modulating the trapping/release of photogenerated carriers and the light-gating effect of heterojunction interface barriers under light stimulation. Notably, the FPAS operates without an external power supply, reducing power consumption and enhancing its similarity to biological receptors. An optical synaptic array was further fabricated by arranging FPAS devices on a polyimide substrate to demonstrate the array’s basic functions of optical sensing and memory storage. The FPAS array can detect and memorize UV irradiation patterns, retaining them in memory over extended periods.

Currently, photonic synapses primarily use linear models to process optoelectronic signals. However, in practical applications, input optoelectronic signals often contain complex nonlinear structural information, such as dynamic illumination and color mixing [136–139]. Traditional neuromorphic devices struggle to handle such nonlinear structural information effectively through linear models, severely impairing the network’s ability to perceive tasks across different spatial and temporal scales [140]. In mammalian neural dynamics, synapses cooperate across multidirectional response scales through mixed weight plasticity, enabling organisms to adapt to nonlinear factors in response to external stimuli [141–143]. For example, axo-axonic synapses—predominantly found in sensory neural inputs—regulate the magnitude of postsynaptic potentials by autonomously adjusting the concentration of neurotransmitters released from the presynaptic membrane. This phenomenon is termed presynaptic homeostatic depression (PHD) [144, 145]. Inspired by this, Gao et al*.* [129] proposed a feedforward light-adaptive hybrid weight plasticity organic neuromorphic transistor by introducing an axon (IDTBT/PVA)-axon (P0FDIID/N2200) structure (Fig. 8b). Owing to the coupling of the space charge potential and gate potential, light excitation and light inhibition occur simultaneously in the channel under constant light intensity interference, allowing the device to transition from a linear to a nonlinear mode via feedforward light adaptation. The device achieves adaptive tone mapping within 5 s for processing static information. Moreover, robust recognition of dynamic information achieves a 261% improvement in recognition accuracy, with accuracy exceeding 90% for 21 types of motion information. This work provides a new strategy for developing advanced neuromorphic devices with great potential in fields such as intelligent driving and brain-inspired computing.

Overall, researchers are continuously constructing OPT-based photonic synaptic devices with higher performance and lower power consumption through various approaches, but there are still some challenging issues to solve with respect to material aspects: (1) the balance between device performance and energy consumption and (2) the compatibility between materials and biological systems. As previously mentioned, the performance of OPTs mainly depends on the carrier mobility of the material, and high carrier mobility implies high energy consumption. Therefore, how to find a perfect balance between the two is a problem that researchers should continue to study in depth. For photonic synapses, one of the most important applications is in simulating artificial vision, and the fabricated photonic synaptic devices face the issue of biological compatibility. Thus, although materials such as perovskites and PbS quantum dots exhibit excellent properties, the toxicity of the heavy metal Pb contained in lead-based organic‒inorganic hybrid perovskites and other similar materials still need to be considered.

## Operational Behavior of Organic Photonic Synapses

The response to light is one of the prerequisites for the operation of organic photonic synapses. An in-depth understanding of the photoresponse behavior and mechanism of OPT-based photonic synaptic devices is one of the most critical prerequisites for constructing photonic synapses with excellent performance and the ability to mimic human synapses. Two fundamental processes are usually involved during photonic synapse operation: the photoresponse (including the photogeneration of charge carriers and transport) [146–150] and the charge storage process [151–154]. The photoresponse behavior emphasizes the effective acquisition of external light information by photonic synapses, whereas the charge storage process is aimed at the storage of signals. The synergistic effect of these two aspects determines the synaptic behavior of the device and the establishment of its synaptic plasticity. In the following, we discuss some recent progress concerning these two processes.

### Photoresponse Process

Under normal conditions, when a phototransistor is stimulated by appropriate external light, the channel current increases, which follows the positive photoconductive response behavior reported in most preceding literature. Recently, the negative photoconductive response, in which the channel current or conductivity decreases upon high illumination, has been frequently reported and has attracted extensive interest. Negative photoconductivity can directly mimic inhibitory synaptic functions, complementing positive photoconductivity and enabling fascinating functionality [157–161]. In the field of photonic synapses, negative photoconductivity serves as a key functional basis for realizing brain-like information processing [162–165]. Specifically:

1. Through the synergistic action of the PPC and NPC, photonic synapses can achieve dynamic enhancement and depression of SW, which forms the basis for constructing neuromorphic systems with learning and memory capabilities. The introduction of the NPC encodes the IPSC, reducing power consumption in the overall synaptic system, inhibiting excessive neuronal activation, and avoiding signal overload.
2. NPC can also enhance the functionality and flexibility of photonic synaptic devices. Currently, reports on NPC, whether based on inorganic or organic materials, can be broadly classified into three types: gate-voltage dependent, wavelength dependent, and light-intensity dependent. Therefore, by applying light stimuli with different gate voltages, wavelengths, or intensities, the PPC and NPC can be independently controlled, enabling the fine-grained programming of synaptic weights. This meets the diverse signal-processing requirements in neuromorphic computing and achieves multidimensional signal processing.
3. From a practical application perspective, when image data are acquired, harsh environmental conditions in real scenes—such as uneven illumination and unstable fields of view—introduce uncertainties that lead to nonideal factors in captured images. These issues increase hardware complexity and reduce image recognition efficiency. Therefore, in addition to PPC, introducing NPC from another perspective and constructing dual-adaptive devices for on-sensor processing are highly important for artificial vision adaptation. This approach enables precise in situ enhancement and filtering of unstructured information, addressing the challenges of dynamic real-world environments.

**Fig. 9:**
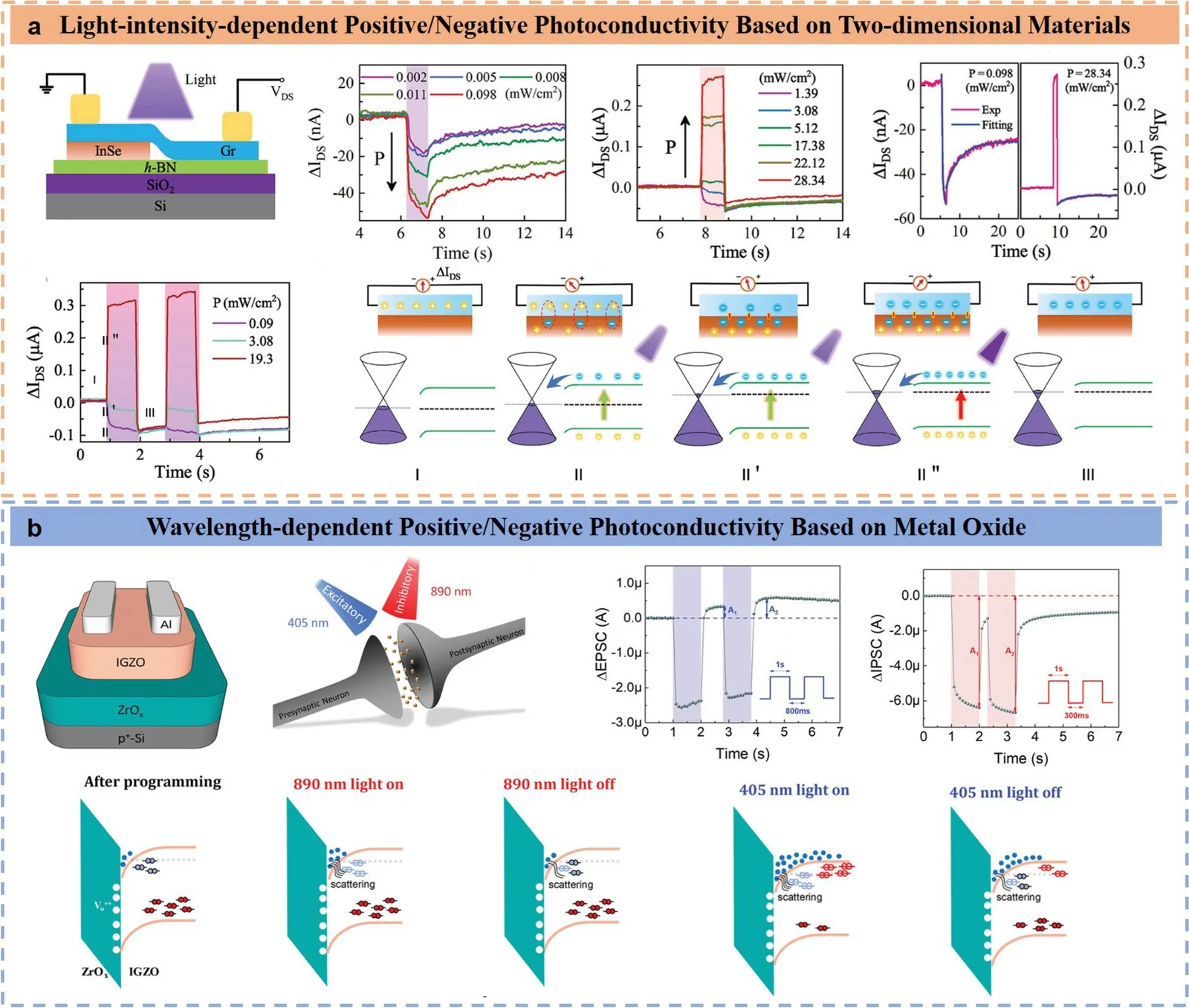
Recent advances and working mechanisms of inorganic negative photoconductance. **a** Light-intensity-dependent positive/negative photoconductivity based on two-dimensional materials. Reproduced with permission [155] Copyright (2024), Wiley–VCH GmbH. **b** Wavelength-dependent positive/negative photoconductivity based on metal oxide. Reproduced with permission [156] Copyright (2023), Wiley–VCH GmbH

**Fig. 10:**
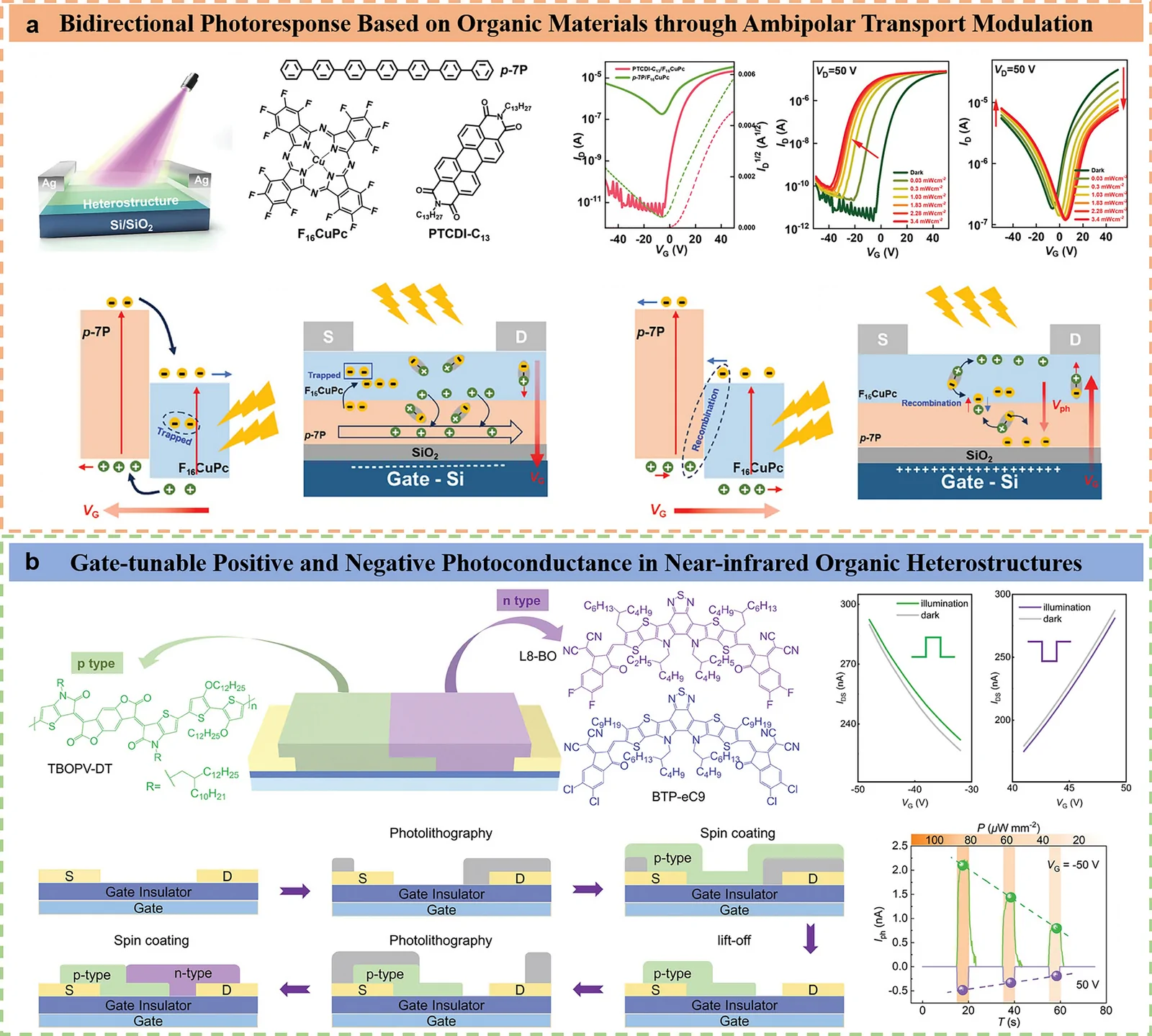
Recent advances and working mechanisms of organic negative photoconductance. **a** Bidirectional photoresponse based on organic materials through ambipolar transport modulation. Reproduced with permission [166] Copyright (2024), Wiley–VCH GmbH. **b** Gate-tunable positive and negative photoconductance in near-infrared organic heterostructures. Reproduced with permission [167] Copyright (2024), Wiley–VCH GmbH

To achieve NPC in OPTs, researchers often utilize bipolar heterostructure channel layers constructed from p-type and n-type semiconductor materials, enabling dual-channel transport of holes and electrons while allowing a controllable bidirectional photoresponse under illumination [168]. However, current reports on NPC are predominantly focused on inorganic semiconductors. For example, Gao et al*.* [155] proposed a graphene/InSe/h-BN heterostructure that exhibits light-intensity-dependent NPC and PPC by adjusting the intensity of a single-wavelength laser (Fig. 9a). Mi et al. [156] used an all-optical IGZO/ZrO_x_ phototransistor to simulate synaptic functions via wavelength-dependent PPC and NPC generated by ionization of neutral oxygen vacancy (V_O_) and metal–metal bonding (M-M) defects in IGZO under visible light (405 and 520 nm) and NIR light (750, 890, and 980 nm) illumination (Fig. 9b). Encouragingly, researchers have also conducted in-depth studies on NPC in organic materials. Xue et al. [166] developed a simple yet effective organic thin-film phototransistor with controllable NPC in the UV band through bipolar transport modulation (Fig. 10a). Xu et al. [167 introduced an organic heterostructure with a strong NIR photoresponse, achieving a PPC and NPC for 1050 nm NIR light by modulating the gate voltage (Fig. 10b). Below, two experimental cases and related mechanisms of NPC in OPT-based photonic synapses are introduced.

**Fig. 11:**
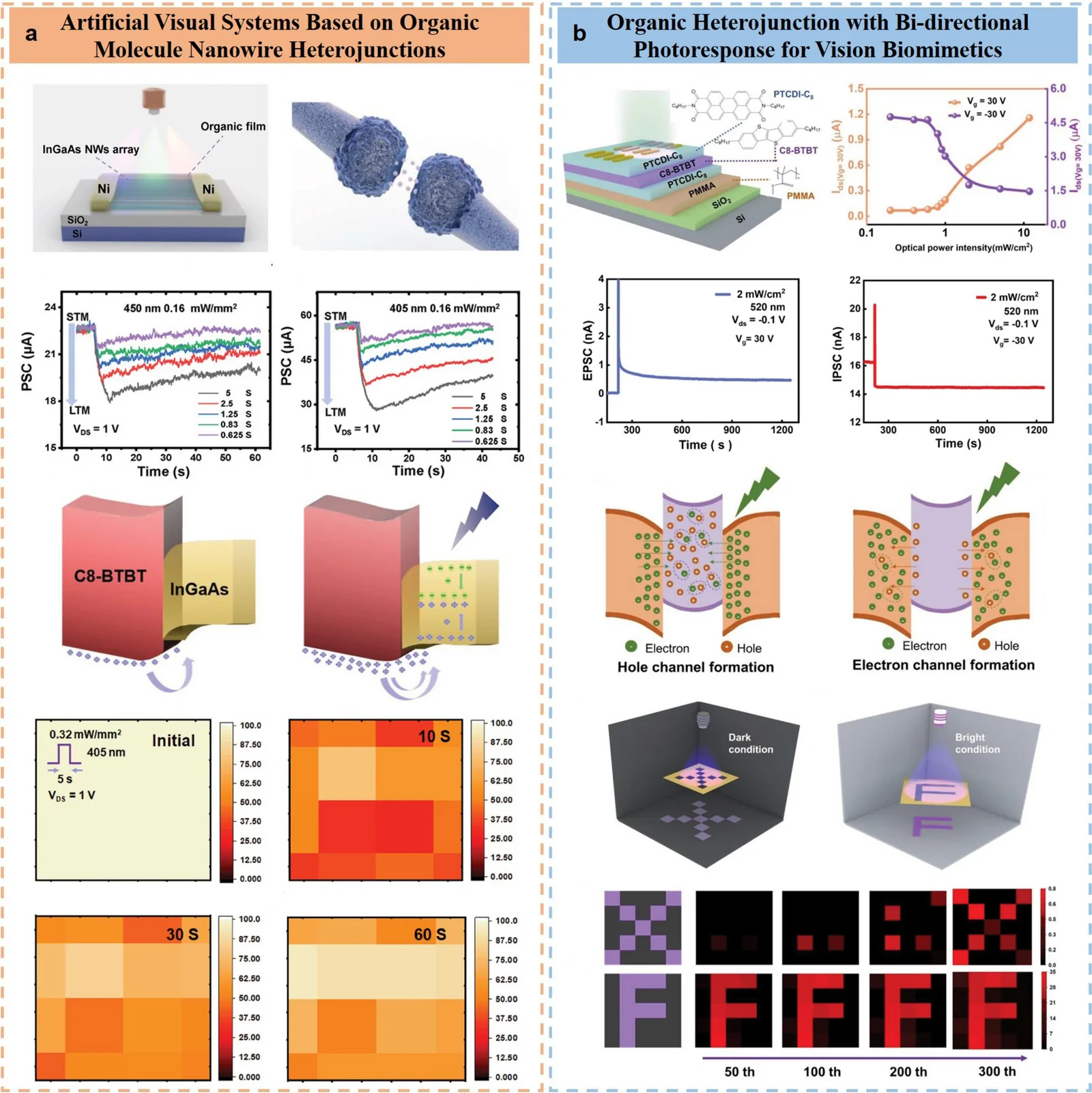
Summary of the negative photoconductance in OPT-based photonic synapses. **a** Artificial visual systems based on organic molecule nanowire heterojunctions. Reproduced with permission [169] Copyright (2022), Wiley–VCH GmbH. **b** Organic heterojunction with bi-directional photoresponse for vision biomimetics. Reproduced with permission [174] Copyright (2024), Wiley–VCH GmbH

In 2023, Xie et al. [169] proposed and demonstrated an artificial photonic synaptic device based on organic molecule–nanowire heterojunctions (Fig. 11a). They encapsulated indium gallium arsenide (InGaAs) nanowire arrays with p-type organic semiconductors of C8-BTBT or n-type OSCs of phenyl-C_61_-butyric acid methyl ester (PC_61_BM) thin films, forming two distinct i-type heterojunctions (i.e., p-n C8-BTBT/InGaAs or n–n PC_61_BM/InGaAs). Owing to the different dominant carriers of the two organic materials, tunable photoconductivity was achieved under irradiation from the ultraviolet to visible light range. The results showed that hole carriers from C8-BTBT or electron carriers from PC_61_BM were injected into InGaAs NWs under light stimulation, leading to persistent NPC or PPC in InGaAs NWs. Owing to the high surface-to-volume ratio of InGaAs NWs, these two heterojunctions easily realize well-defined synaptic functions. Notably, synaptic behaviors, including the STP, LTP, EPSC, IPSC, and STP-to-LTP transitions, were reliably demonstrated under visible and ultraviolet lasers (including the solar-blind range). Owing to differences in Fermi levels, a built-in electric field forms at the InGaAs-C8-BTBT interface, causing band bending without applied light pulses. Additionally, the dominant carriers in the p-type C8-BTBT film are holes, which transfer to the InGaAs NWs and suppress current. Moreover, the large energy gap and potential barrier between the LUMO level of C8-BTBT (− 1.8 eV) and the CB of InGaAs make electron transfer from C8-BTBT to InGaAs NWs more difficult than hole transfer. Moreover, C8-BTBT exhibited stronger absorption in the UV range. When short-wavelength light pulses irradiate the heterojunction, more holes generated from C8-BTBT are injected into the NWs because of the built-in electric field at the InGaAs-C8-BTBT interface. The injected holes recombine with electrons in the NWs, inducing the NPC phenomenon. Compared with single NW-based devices, the integration of printed NW arrays and solution-processed organic materials has great potential as an active material for large-area neuromorphic visual networks. Under *V*_G_, a prototype 4 × 4 artificial photonic synaptic device array with optical memory functions was demonstrated. Moreover, two photonic synaptic devices with tunable photoconductivity were used to construct a hardware core simulating human visual processing and recognition. Different hardware cores can detect distinct features in images, similar to the receptive fields of the human retina. These features were then input into a neural network to classify six letters of different colors, achieving an impressive 100% classification accuracy—compared with only 51% without the hardware core.

Currently, research on the photoresponse of organic materials has focused mainly on PPC, with fewer studies on NPC and even fewer studies on the coexistence of both PPC and NPC in the same device. The relevant physical mechanisms still need to be explored [25, 170]. Additionally, optoelectronic devices with bidirectional photoresponse capabilities have unique advantages and great application potential in visual adaptation simulations [171–173]. The development of suitable reconfigurable organic optoelectronic devices with both PPCs and NPC is expected to be applied in bioinspired visual systems, promoting the rapid development of brain-like visual bionic systems. Thus, in 2024, Shi et al*.* [174] constructed an organic heterojunction optoelectronic device with a sandwiched channel structure (Fig. 11b). The device exhibits excellent bipolarity, with a current on/off ratio (*I*_on_/*I*_off_) greater than 10⁶ in the transfer curve during hole transport. Under illumination, by adjusting *V*_G_, two distinct light-controlled modes—PPC and NPC—can be achieved in a single device. Typical synaptic behaviors such as EPSC, IPSC, STP, and LTP were also simulated. The bottom n-type PTCDI-C_8_ layer provides a conduction pathway for electron transport, the middle p-type C8-BTBT layer serves as a pathway for hole transport, and the top n-type PTCDI-C_8_ layer offers an additional pathway for electron transport. When the organic triple-layer heterojunction optoelectronic device is exposed to 520 nm light, the n-type semiconductor PTCDI-C_8_ film generates many photogenerated carriers, whereas C8-BTBT shows almost no response to 520 nm light owing to its wide bandgap. During hole transport, abundant electrons in PTCDI-C_8_ are transferred to the C8-BTBT hole transport layer, recombining with holes there and reducing the overall hole count, leading to NPC. At a voltage of 30 V, electrons become the dominant carriers. When simultaneously exposed to 520 nm light stimulation, a small number of photogenerated holes in the C8-BTBT semiconductor layer are conducted to the PTCDI-C_8_ layer. However, these holes struggle to recombine with the abundant electrons generated in the PTCDI-C_8_ layer, causing the device to exhibit a PPC. Finally, they constructed an array of organic heterojunction phototransistors to achieve adaptive regulation of the human retina under different brightness environments.

### Charge Storage Process

One of the important characteristics of photonic synapses is the realization of storability, which is the key to achieving synaptic plasticity. In OPT-based photonic synapses, carriers in the channel are regarded as information. The generation or erasure of information can be accomplished via the PPC or NPC mentioned in the previous sections. After the photonic synaptic device is stimulated by light, the photogenerated excitons dissociate into photogenerated electrons and photogenerated holes. Under the action of the bias voltage, they are captured by the source/drain electrodes, forming an external current. During the movement of photogenerated electrons and holes to the source/drain electrodes, these charges can be trapped and stored by trap states or interface states, which constitute the fundamental basis and core origin of the charge storage process. Trap states can be located in the bulk phase or at the interface of the semiconductor material, and they can temporarily bind electrons or holes to form charge storage. Interface states exist in the interface region of different materials and can also play a similar role in charge storage [175].

**Fig. 12:**
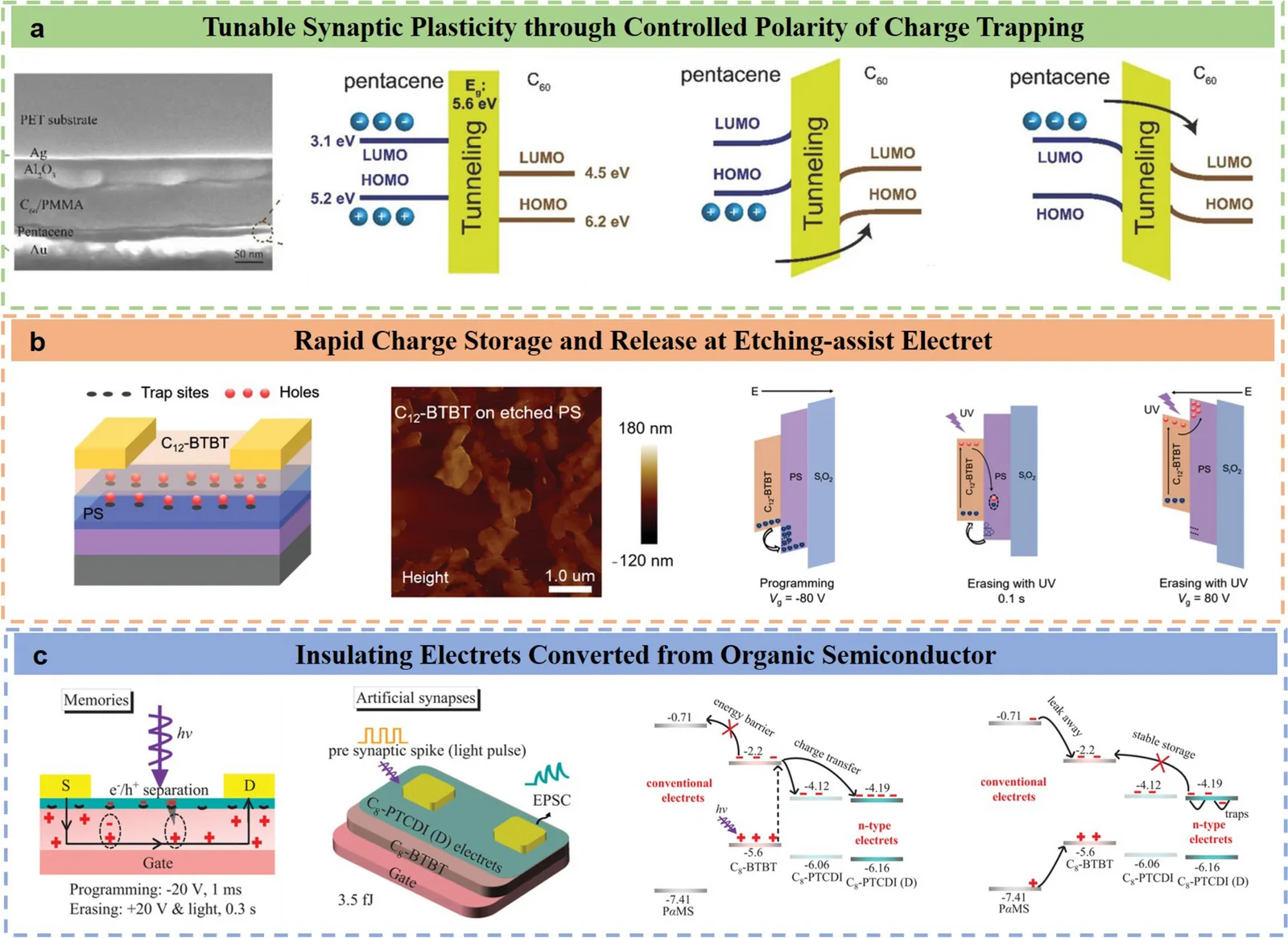
Summary of charge storage behavior in OPT-based photonic synapses. **a** Tunable synaptic plasticity through controlled polarity of charge trapping. Reproduced with permission [176] Copyright (2018), WILEY‐VCH Verlag GmbH & Co. KGaA, Weinheim. **b** Rapid charge storage and release at etching-assist electret. Reproduced with permission [177] Copyright (2022), Wiley‐VCH GmbH. **c** Insulating electrets converted from organic semiconductor. Reproduced with permission [151] Copyright (2023), Wiley‐VCH GmbH

To enhance the charge trapping performance, a charge storage layer can be employed between the channel material and the dielectric layer. Most of the experimental cases introduced earlier adopted this structure. Additionally, Ren et al. [176] demonstrated gate-tunable synaptic plasticity through controlled polarity of charge trapping in fullerene composites (Fig. 12a). C_60_ trapping sites are doped in PMMA by a facile solution process to form a hybrid structure. Electrets are commonly used charged insulators that generate a quasipermanent electric field. However, insulators intrinsically exhibit high electrical resistance; thus, charge injection and release inside insulators typically require high voltages or long bias times, limiting applications that require high writing/erasing speeds. Qiao et al. [177] introduced the etching-assisted insulator polymer polystyrene (PS) to realize rapid charge storage and release (Fig. 12b), which selectively induces a high density of charge traps at the top of C_12_-BTBT for flash-type memories, photodetectors, and artificial synapses. In addition, when conventional electrets come into direct contact with semiconductors, the energy level mismatch at the interface results in a low memory speed and high energy consumption of electret devices because both charge injection and storage are nonconductive. To address this, Li et al. [151] converted the n-type semiconductors N, N & PRIME, i.e., dictyl-3,4,9,10-perylene tetracarboxylic diimide (C_8_-PTCDI), to C_8_-PTCDI (D) via oxygen degradation for high-performance transistors, memories, and artificial synapses (Fig. 12c). The synthesized C_8_-PTCDI (D) electrets, upon charging via an electric field and/or illumination, maintain the energy levels characteristic of n-type semiconductors, and this preservation of energy levels enables efficient charge trapping, as the specific electronic structure of n-type semiconductors under these charging conditions creates favorable sites for the capture and retention of charges.

The static charges trapped in the storage layer can manifest as a semipermanent electric field and/or a dipole moment. Dielectrics with strongly polar functional groups can induce strong shallow traps, which are often used as charge storage layers. To achieve a longer-term memory function, shorter side chains are often used, resulting in stronger deep traps [172]. However, trap states may lead to increased charge recombination, thereby reducing the efficiency and performance of the phototransistor. Interface trap states require precise control of the interface morphology, so it is difficult to achieve high reliability and repeatability. They may also affect the response speed and stability of the device. This is a trade-off that needs to be considered when constructing photonic synapses.

Achieving long-term plasticity in OPT-based synapses requires persistent modulation of the channel conductance. Beyond classical charge-trapping, several emerging strategies have recently demonstrated remarkable memory retention. Park et al*. *[178] incorporated porous or ionic pathways enables physical confinement of mobile ions to maintain long-lived excitatory states. Lee et al*.* [179] have reported that ferroelectric coupling offers an alternative approach, where polarization switching can directly regulate synaptic weights in a non-volatile manner. In addition, multi-layer charge-storage architectures and optimized heterojunction potentials improve the stability of trapped carriers against recombination [180]. These advances provide promising pathways toward reliable LTP necessary for high-level neuromorphic learning.

Benefiting from the diverse material systems and tunable operating characteristics described above, organic photonic synapses have demonstrated significant potential across a range of cutting-edge applications. In the following section, we will delve into the recent advances and emerging applications of organic photonic synapses in various fields.

## Frontier Applications and Advances in Organic Photonic Synapses

Driven by recent advances in organic photonic synapses—particularly interfacial exciton manipulation, bias-gated neuromodulation, and bio realistic kinetic emulation—novel neuromorphic applications have rapidly proliferated. Notable implementations that integrate in-sensor convolutional processing, spike-timing-dependent plasticity (STDP) engines, and optically reconfigurable neural networks are paving the way toward von Neumann architecture-free intelligent systems. Inspired by the human eye and biological synapses, one of the most natural and promising applications of photonic synapses is simulating artificial vision, including recognition of various visible light wavelengths and adaptive adjustment to light brightness similar to that of the human eye [72, 73, 181–183]. However, in practical applications, human vision has inherent limitations, such as the inability to detect polarized light, recognize light signals outside the visible spectrum (e.g., ultraviolet or infrared), or accurately identify objects in extremely low light. These limitations have prevented photonic synapses from leveraging polarization, UV, or IR information to improve object recognition/tracking, thus restricting advancements in intelligent applications such as facial recognition [184–186], visual prosthetics [187–189], autonomous navigation [190–192], and surveillance systems [193, 194]. Driven by extensive research, organic photonic synapses have now achieved fundamental pattern recognition capabilities such as mobile object recognition [75], handwritten digit recognition [76], and image recognition [77, 128]. Building on this foundation, they have further evolved to enable diverse applications including Morse code sensing [99], visual adaptation [100], night imaging [105], high-dimensional storage [113], polarization sensitivity [195, 196], fused imaging [197], and in-sensor computing [198]. Additionally, with advancements in fabrication processes, photonic synapses are gradually being integrated at large scales [199, 200]. Therefore, this section introduces recent technological applications and advances in photonic synapses, including experimental cases of polarization-sensitive devices, biomimetic synapses, synaptic security devices, dynamic/static image recognition, and large-area lithography-fabricated synapses.

### Polarization-Sensitive Organic Photonic Synapses

Polarization-sensitive neuromorphic visual systems represent highly integrated multifunctional platforms that combine polarization detection, learning, memory, and processing [201, 202]. Specifically, polarization-sensitive elements serve as the core for polarization detection, with materials such as liquid crystals, supramolecular polymers, and molecular materials developed to realize this function. However, these materials often lack charge transport capabilities, requiring the integration of optoelectronic functional layers into heterostructures to form stacked diodes or transistors—leading to complex fabrication processes and suboptimal performance [203, 204]. To address these challenges, hybrid organic‒inorganic perovskites (HOIPs) have emerged as promising candidates because of their solution processability, tunable bandgap, high absorption coefficients, and high carrier mobility [205–207]. Moreover, organic semiconductor single crystals, with unique advantages such as abundant availability, solution processability, flexibility, and tunable optoelectronic properties, are emerging in this field [208, 209]. These materials have achieved notable breakthroughs in terms of high linear dichroic ratios (LDRs), high photosensitivity, and diverse response wavelength bands, showing vigorous development momentum.

**Fig. 13:**
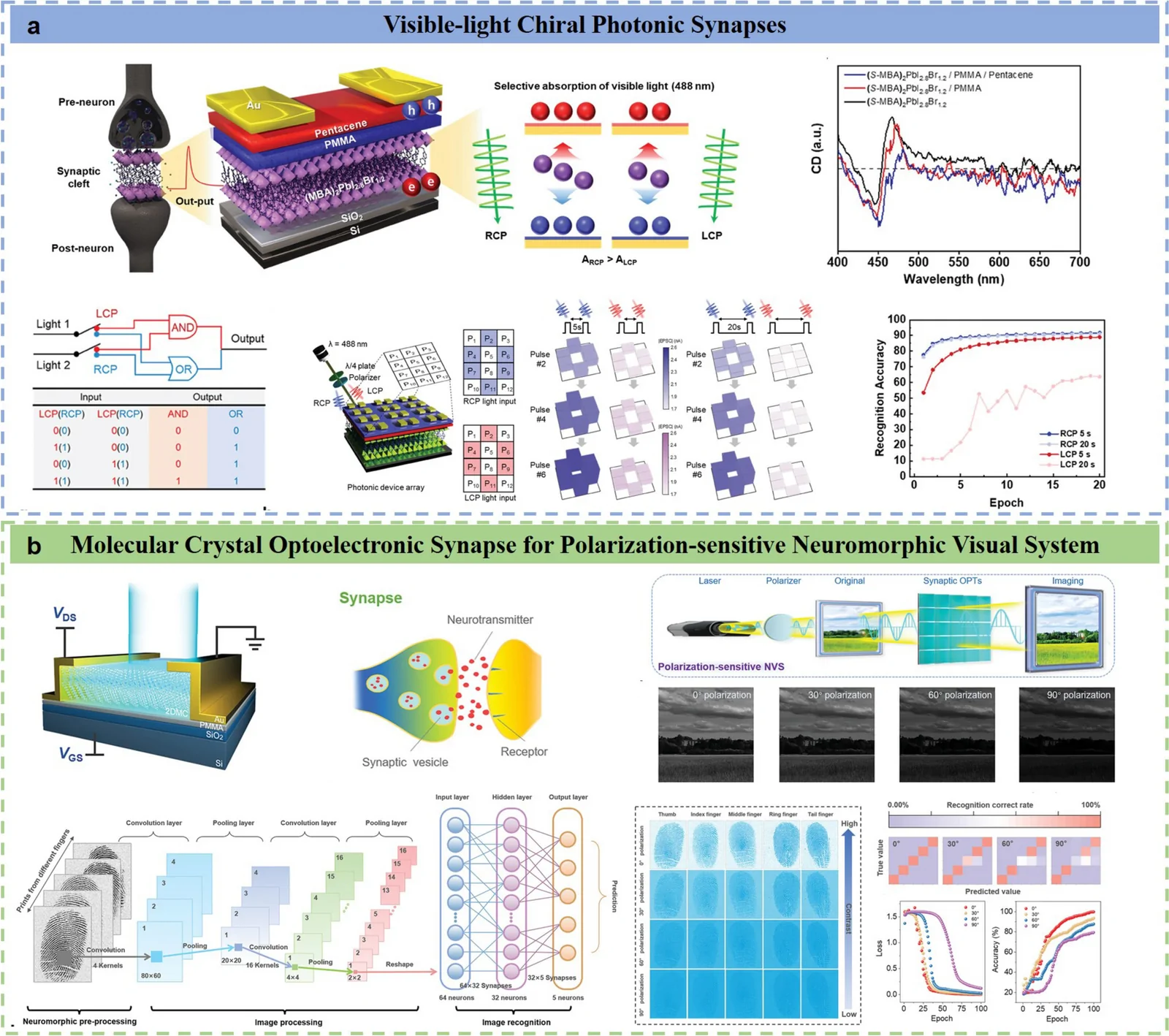
Advanced applications of organic photonic synapses in polarization detection. **a** Visible-light chiral photonic synapses. Reproduced with permission [196] Copyright (2024), Wiley–VCH GmbH. **b** Molecular crystal photonic synapse for polarization-sensitive neuromorphic visual system. Reproduced with permission [195] Copyright (2024), Wiley–VCH GmbH

Hwang et al*.* [196] demonstrated a synaptic device based on two-dimensional (2D) chiral hybrid organic–inorganic perovskites of methylbenzylamine (MBA), which can absorb visible circularly polarized light (CPL) (Fig. 13a). The device features a vertical structure: Si/SiO_2_/(R, S-MBA)_2_PbI_2.8_Br_1.2_/PMMA/pentacene/Au source–drain electrodes. Circular dichroism (CD) measurements and photoluminescence (PL) analysis confirmed that these heterostructures are suitable for the selective detection of visible CPL. The heterojunction between the chiral HOIPs and the pentacene layer induces CPL direction-dependent photocurrent generation and charge separation, enabling memory characteristics that demonstrate chirality-related synaptic properties. The device exhibited a photocurrent asymmetry factor as high as 0.3 and a photoresponsivity of 130 mA W^−1^. Additionally, the system demonstrates programmable and erasable processes via chiral optical and electrical control, respectively. This device not only mimics the synaptic properties of existing photonic synaptic devices, but also exhibits distinct synaptic behaviors depending on the CPL. On the basis of the chiral perovskite heterostructure, they achieved simple logical operations using two pairs of light sources for the first time. Furthermore, as the pulse interval time increases, the device shows improved discrimination between right-circularly polarized (RCP) and left-circularly polarized (LCP) light. Moreover, different CPL types and pulse intervals can induce differences in the pattern recognition process. Simulations of polarization-related image recognition via an ANN show that filtering images based on the CPL direction and pulse interval time can affect the network’s recognition accuracy. By introducing 2D chiral HOIPs into traditional optoelectronics, this work provides a facile approach for realizing visible-light CPL-modulated systems and developing polarization-sensitive neuromorphic (PNS) technologies for neuromorphic visual systems.

Polarization detectors based on organic semiconductor single crystals lack synaptic functions characterized by excellent conductivity tuning and controllable memory capabilities. This limitation arises from the prevalent use of multilayer single crystals, where interlayer screening effects hinder precise conductivity control by photogating [170, 210, 211]. As a result, these devices typically exhibit persistent photoconductivity or fast photoresponse characteristics rather than finely tuned conductivity modulation, which is indicative of synaptic behavior. Constructing an integrated neuromorphic device that seamlessly combines all these functions and demonstrates holistic superior performance thus remains a formidable challenge. Dong et al*.* [195] pioneered the development of a linearly polarization-sensitive neuromorphic visual system using few-layer two-dimensional molecular crystals (2DMCs), achieving integrated functionality and outstanding overall performance (Fig. 13b). The 2-decyl-7-phenyl-[1]benzothieno[3,2b][1] benzothiophene (Ph-BTBT-10) compound was selected as the photoactive channel layer owing to its unique anisotropy, remarkable carrier mobility, and excellent air stability. Its 2DMC configuration offers several notable advantages for constructing polarization-sensitive synaptic OPTs: long-range periodic ordering induces intrinsic anisotropy, leading to significant anisotropic absorption and transport properties. This establishes a foundation for exploring the linear dichroism inherent in polarized light. The ultrathin nature of 2DMCs ensures a uniform carrier concentration within the channel and enables consistent modulation. In contrast, exposed carrier transport channels directly couple with polarized light, enhancing light perception. By comparison, multilayer crystals exhibit uneven carrier concentration distributions, as electric gates can only control a few molecular layers at the dielectric/semiconductor interface, leaving photogenerated carriers in the top molecular layers unmodulated. The introduction of a mechanism for trapping photogenerated carriers in 2DMCs enables controllable memory tuning across a wide current range, facilitating the emergence of unique optoelectronic synaptic properties—including a high dynamic modulation range and polarization-angle-dependent sensitive responses. Leveraging these advantages, the synaptic OPT demonstrates holistic superior performance: a leading LDR of up to 3.85, excellent carrier mobility (μ) of 9.8 cm^2^ V^−1^ s^−1^, a high photoresponsivity (R) of 1.47 × 10^4^ A W^−1^, and precise emulation of various synaptic functions. Furthermore, the evolved polarization-sensitive neuromorphic visual system achieves exceptional visual polarization imaging, revealing complex polarization details of objects across multiple dimensions, and demonstrates a remarkable 99.8% recognition accuracy in noncontact fingerprint detection—highlighting its enormous potential in advanced intelligent sensing systems.

### Bionic Design of Organic Photonic Synapses

State-of-the-art artificial visual systems are less sophisticated than their biological counterparts in terms of structural simplicity, self-regulation, and versatility. For example, light-adaptive devices and neuromorphic phototransistors either employ complex multilayered designs or integrate detectors and processors, increasing manufacturing costs and complexity [134, 212, 213]. Integrating multiple functions into a single, integrated unit remains a key challenge.

**Fig. 14:**
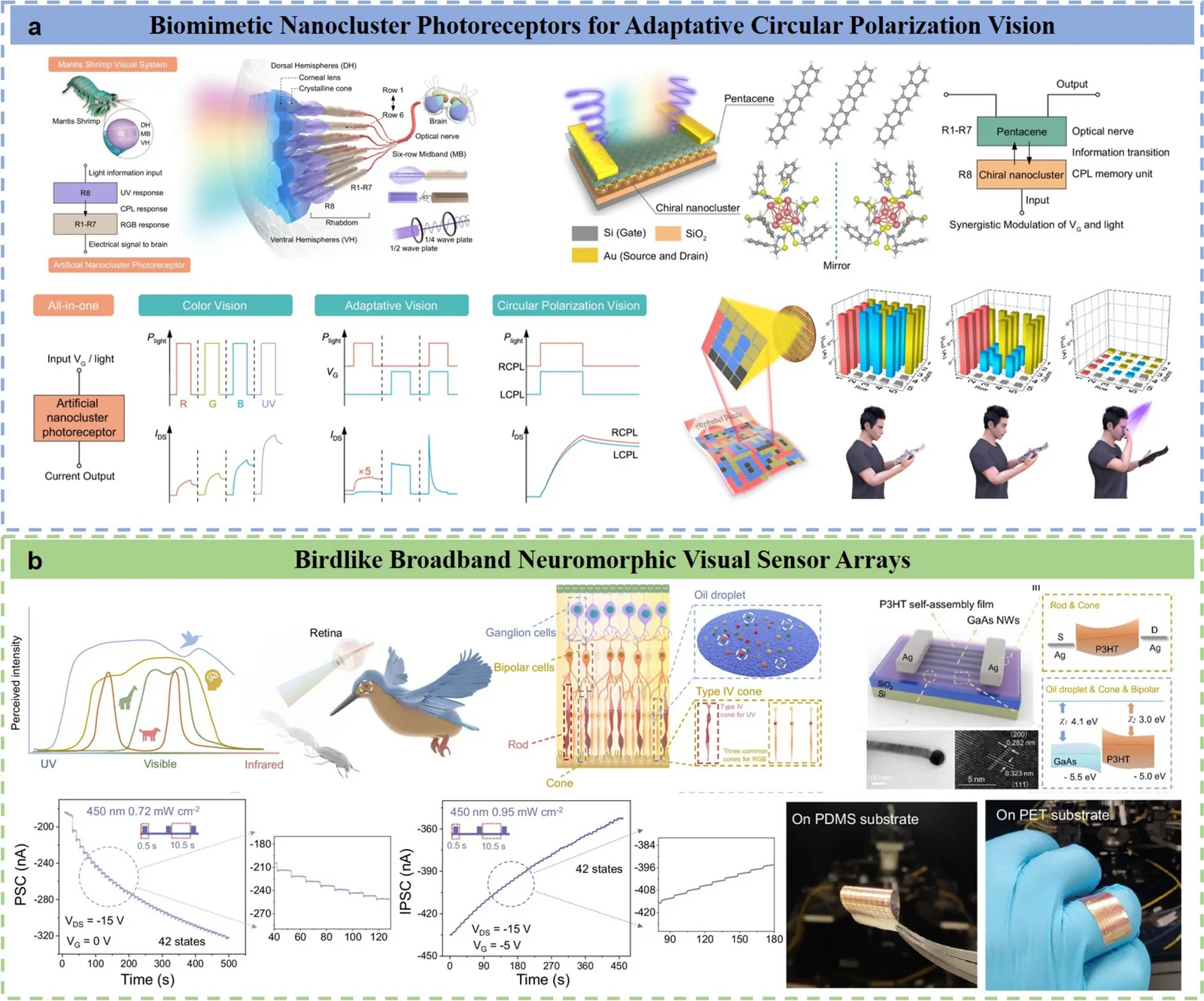
A summary of recent biomimic organic photonic synapses. **a** Biomimetic nanocluster photoreceptors for adaptative circular polarization vision. Reproduced with permission [219] Copyright (2024), Spring Nature. **b** Birdlike broadband neuromorphic visual sensor arrays [197] Copyright (2024), Spring Nature

The visual system of the mantis shrimp is equipped with 16 photoreceptors, enabling it to perform multiple tasks, such as color recognition, adaptive vision, and CPL perception [214]. Although these functions have been individually achieved via full-color absorbing materials [215], circularly polarized molecular assemblies [216, 217], chiral compounds [218], or light-adaptive devices, developing bionic hardware capable of parallel processing for color recognition, tunable adaptation, CPL sensing, and polymorphic readout holds significant theoretical and practical value. Inspired by mantis shrimp, Wen et al*.*  [219] demonstrated an artificial cluster photoreceptor (ACP) array based on heterostructures formed by chiral nanoclusters and organic semiconductors (Fig. 14a). The light-assisted, tunable Fermi levels of nanoclusters act as electron reservoirs, precisely controlling the trapping and release of charge carriers in the proposed artificial photoreceptors. Visual adaptation in ACPs is achieved by regulating photogenerated carriers at the interface between organic molecules and Ag nanoclusters, with wavelength- and intensity-dependent adaptability further validated through shape and color recognition. Leveraging the chirality of Ag nanoclusters, ACPs can distinguish CPL light, closely mimicking the mantis shrimp’s CPL perception function. To further explore the light-valence mechanism of silver nanoclusters, the charge storage locations and transfer pathways were investigated via transient absorption and photoluminescence techniques. In summary, the ACP emulates the mantis shrimp’s complex visual system, integrating full-color adaptation and circular polarization vision through a simple architecture. From a practical perspective, this bionic system can be upgraded to meet the needs of vision-related AI hardware and encrypted information communication.

In biological vision, birds have long been a fascinating subject in nature, sparking significant interest in bionics. Enhancing device perception through animal bionics is an effective approach to achieve more robust neuromorphic sensing [220, 221]. When combined with fused imaging technology, a single device can perceive, integrate, and compute multiple signals from different sources, displaying them in a single image or video. Xie et al*.* [197] proposed a dual-junction-enhanced avian broadband neuromorphic visual sensor (BBNVS) array with a response range from solar-blind to near-infrared (Fig. 14b). Birds, as natural aerial hunters, possess an unparalleled visual-perception system in nature. To mimic the functional components of avian vision—distinct from human vision—the authors introduced type II van der Waals (vdWs) heterojunctions and Schottky junctions. Specifically, self-assembled P3HT organic films with ordered edge stacking were transferred onto one-dimensional GaAs nanowire arrays to construct p-n junctions in the out-of-plane direction. A Ag source and drain electrodes were then applied to form Schottky junctions. These two functional structures effectively emulate the roles of four cone cells, rod cells, bipolar cells, and oil droplets in the avian visual system. Owing to the highly ordered structure of self-assembled P3HT molecules and the Schottky barrier, BBNVS exhibits exceptional broadband memory sensing and computing performance. It achieves over 5-bit memory sensing and computing processes under blue and green wavelengths, as well as the ability to handle over 128 memory states in the solar-blind range. A persistent NPC with over 42 memory states is also realized under negative gate voltage modulation. In nature, predators enter an "energy-saving mode" to maintain environmental perception when food is scarce; accordingly, visual enhancement in BBNVS is reflected not only in non-volatile broadband response but also in low-power sensing capability. The proposed BBNVS mimics avian visual behavior under zero gate and source–drain voltages, operating in self-powered mode with nearly zero power consumption for synaptic devices. To meet the needs of future wearable devices, neuromorphic visual sensor arrays can be fabricated on various substrates, including glass, PET, PDMS, polyimide (PI), and acrylic plates. The excellent bend resistance of chain polymers and stress relief of nanoscale nanowires at bent interfaces ensure that the device maintains robust performance on flexible substrates, even when folded. Finally, a multitask RC system is used to achieve multimodal recognition of moving objects, including shape, motion, color, and ultraviolet grayscale information, with a color recognition accuracy of up to 94%. By integrating broadband sensory enhancement, BBNVS combined with the RC system accomplishes ultraviolet–visible light-fused imaging through multidimensional information integration, recognizing images from ultraviolet and visible cameras simultaneously. This work presents a promising material synthesis and codesign strategy for broadband non-volatile sensing, wearable, and efficient optoelectronic neuromorphic systems with multitask memory sensing and computing capabilities.

### Synaptic Security Devices and Dynamic/Static Image Recognition

**Fig. 15:**
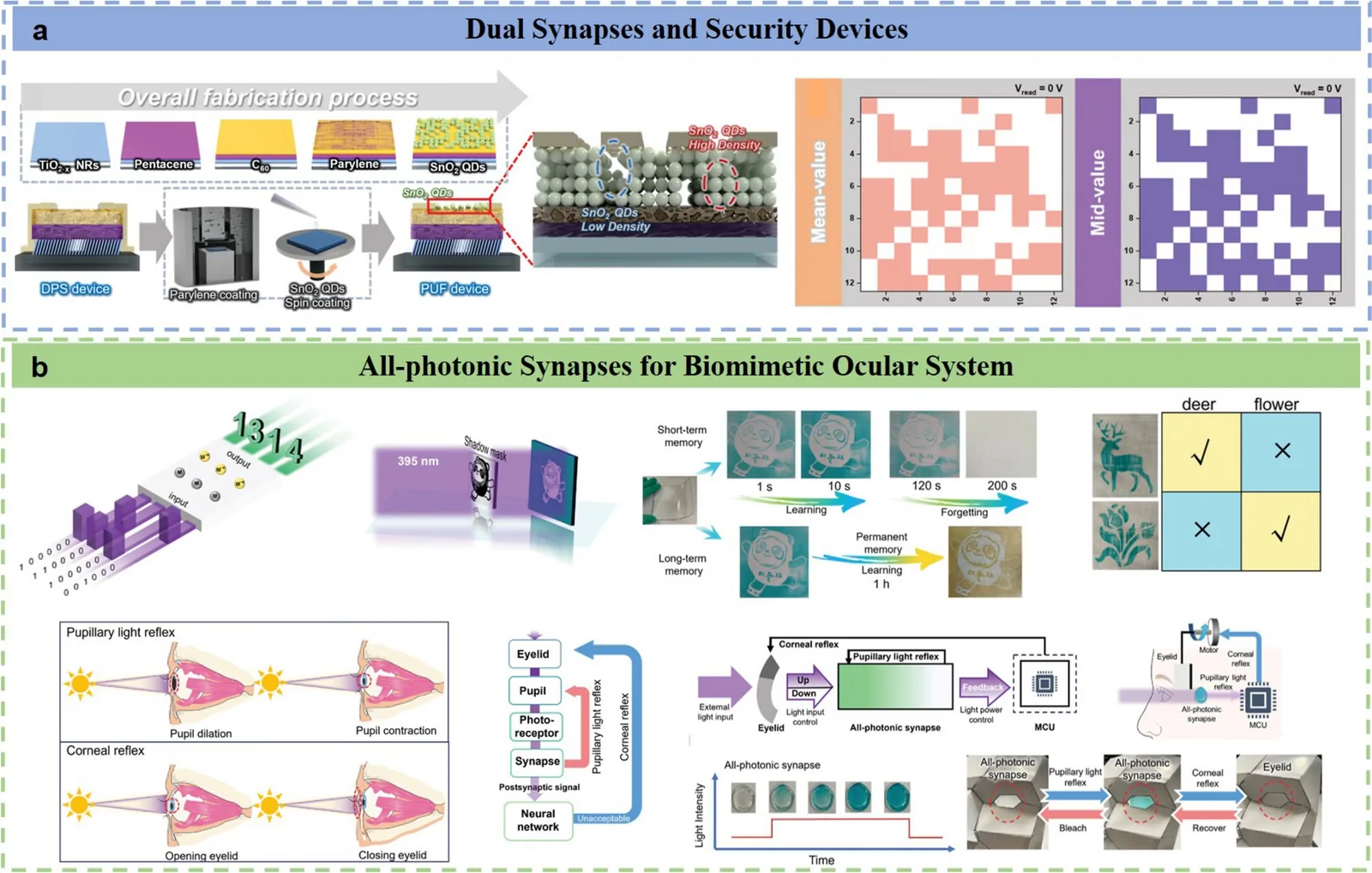
Other advanced applications of organic photonic synapses. **a** Dual synapses and security devices. Reproduced with permission [224] Copyright (2024), Wiley—VCH GmbH. **b** All-photonic synapses for biomimetic ocular system. Reproduced with permission [225] Copyright (2024), Wiley—VCH GmbH

In the context of information explosion, neuromorphic and hardware-based security devices have received significant attention because of their ability to facilitate parallel computing and resist hacking [222, 223]. Jeon et al*.* [224] demonstrated dual-photo synapse (DPS) and physical unclonable function (PUF) devices featuring a ternary TiO_2-x_ NR/pentacene/C_60_ heterostructure (Fig. 15a). For the DPS device, the ternary heterostructure results in DPS characteristics for parallel operation: i) wavelength-dependent (WD) synapses and ii) stimulus-moment-dependent (SMD) synapses. Each synaptic feature exhibits significant plasticity, implying a smooth transition from STP to LTP. Additionally, facial recognition evaluation reveals an 83.3% recognition rate for both operating modes. Notably, the DPS device maintains stable performance on business cards even after a rigorous 49-day testing period and 1000 bending cycles. For the PUF device, a security key with a unique pattern can be created because of the random current distribution caused by the position-dependent doping effect of the SnO_2_ QDs. Moreover, all encryption keys have been verified for near-ideal Hamming distance (inter-HD), uniformity, and entropy. These findings introduce the groundbreaking concept of photon synapses and their successful integration onto various substrates, including hardware-based secure PUF devices.

The human visual system, which processes real-time light signals and protects against excessive illumination, serves as a model for artificial visual systems. Although optoelectronic synaptic devices have been widely used in such simulations, their complex circuitry and high energy consumption have hindered further development [226, 227]. Zhang et al*.* [225] constructed a bionic eye system using organic all-photonic synapses to achieve sensing, memory, processing, and protective light reflex capabilities (Fig. 15b). By performing facial tuning of the synaptic performance through simple molecular engineering, a record 430% PPF value was achieved. Impressively, a large-area (400 cm^2^) and highly uniform (96%) synaptic device demonstrated the four basic functions of the eye system for the first time, greatly simplifying the circuit system and reducing energy consumption. This work provides a facial strategy for constructing all-photonic synapses, which has great potential in prosthetics and neuro-robotics.

### Large-Area Lithography Fabrication of Organic Photonic Synaptic Arrays

With the rapid development of information technology, there is an increasing demand for higher integration density in organic imaging chips to handle the surge in signal volume [228, 229]. Inorganic materials, such as CMOS chips, have achieved very large-scale integration (VLSI) but exhibit poor mechanical compatibility with biological systems and flexible devices [230, 231]. Organic imaging chips can address this issue and are gaining popularity in innovative applications such as stretchable displays, wearable devices, and artificial visual systems [232]. However, owing to the lack of precisely designed nanostructures in semiconductor layers, the application of organic imaging chips has achieved only limited success in large-area fabrication or higher levels of integration.

**Fig. 16:**
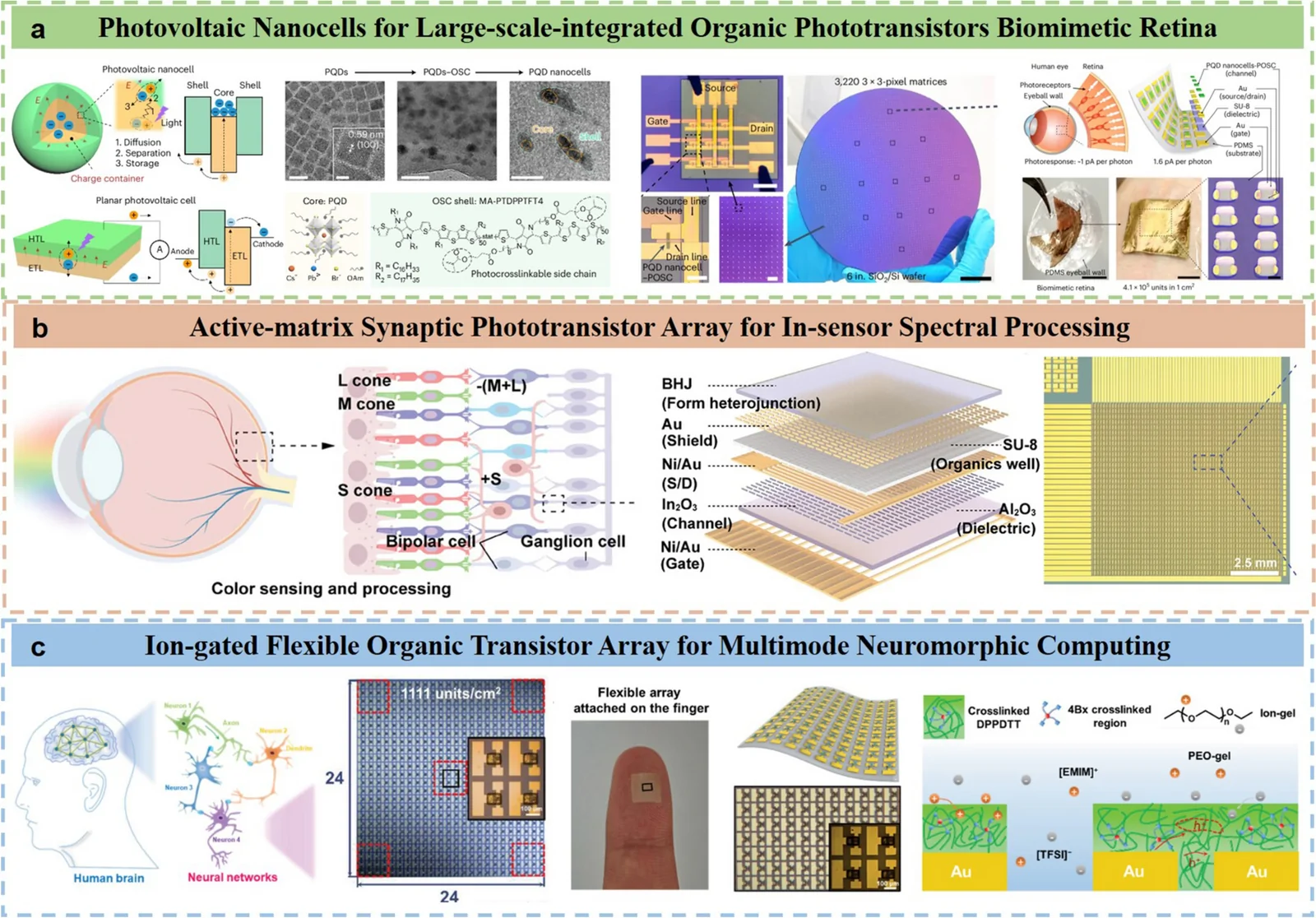
Examples of large-scale photonic synapse fabrication. **a** Photovoltaic nanocells for large-scale-integrated organic phototransistors biomimetic retina. Reproduced with permission [200] Copyright (2024), Spring Nature. **b** Active-matrix synaptic phototransistor array for in-sensor spectral processing. Reproduced with permission [198] Copyright (2024), Wiley—VCH GmbH. **c** Ion-gated flexible organic transistor array for multimode neuromorphic computing. Reproduced with permission [199] Copyright (2024), Wiley—VCH GmbH

Zhang et al*.* [200] demonstrated a photovoltaic-nanobattery enhancement strategy using zero-dimensional optoelectronically active nanomaterials to increase the performance of large-scale integrated (LSI) organic imaging chips (Fig. 16a). They constructed photovoltaic nanobatteries (PQD nanobatteries) with PQD cores and OSC shells and then embedded these PQD nanobatteries into lithographic OSCs (PQD nanobattery-POSCs) to fabricate OPTs. Through lithography, they produced a 2.67-inch full-frame ultra large-scale integration (ULSI) chip with 27 million interconnected pixels. The pixel density of 3.1 × 10^6^ units cm^−2^, equivalent to 4,016 pixels per inch (PPI), reaches the level of the latest commercial full-frame camera chips (Nikon Z8). Owing to increased light absorption, enhanced photoelectric conversion, and charging of numerous photogenerated electrons by PQD nanobatteries, additional in situ photogating modulation and increased photocurrent are achieved. Thus, under performance trade-offs, R > 6 × 10^6^ A W^−1^ and D* > 10^13^ Jones are achieved with small feature sizes, high yields, and low variations. The PQD nanobattery-POSC was further used to fabricate a high-resolution bionic retina for neuromorphic imaging, demonstrating flexibility, resolution (2,318 PPI), responsivity (1.6 pA per photon), and low power consumption (2.7 fJ per spike) comparable to those of biological retinas—showing the potential of LSI organic imaging systems to outperform state-of-the-art technology.

The human retina perceives and pre-processes the spectral information of incident light, enabling rapid image recognition and efficient color adaptation. In contrast, current CMOS image sensors struggle to achieve parallel spectral pre-processing and temporal information fusion, requiring complex circuits, frequent data transmission, and color filters [233]. To address this, Li et al*.* [198] proposed a simple approach to achieve color opponent processing through dual photogates in metal-oxide/organic heterojunctions and demonstrated an active-matrix synaptic phototransistor array (AMSPA) for filter-free dynamic imaging with in-sensor spectral processing capabilities (Fig. 16b). The hybrid phototransistors exhibit bidirectional synaptic photoresponses based on specific input wavelengths, featuring UV-induced excitation and visible/NIR-induced inhibition. Additionally, synaptic phototransistors can mimic the color adaptation process and display a high current dynamic range exceeding 90 dB, significantly resolving the low dynamic range issue typical of bidirectional synaptic devices. Finally, a 32 × 64 AMSPA (128 pixels per inch) was realized via a single-switch transistor and single-synaptic phototransistor (1-T-1-PT) structure, enabling spatial color enhancement and temporal trajectory imaging. These results demonstrate the feasibility of the AMSPA for constructing artificial visual systems.

Current advancements in hardware neuromorphic computing systems are hindered by the difficulty of codesigning high-density artificial synaptic arrays and affordable algorithms [234]. In terms of arrays, extensive research has been conducted to develop high-density two-terminal synapses [126, 235–237], which benefit from their simpler crossbar structure and more mature integration technology [238]. However, owing to the unstable weight update behavior caused by structural defects, two-terminal synapses are not suitable for constructing repeatable hardware neural networks [239]. Additionally, in regard to flexible electronics (a key component of the abovementioned vision), the mechanical flexibility of devices is indispensable [240]. Organic ion-gated transistors (OIGTs) have shown impressive performance in multifunctional neuromorphic systems. However, owing to the vulnerability of organic materials to organic solvents, there is still a lack of efficient and reliable all-lithography methods for scalable manufacturing of high-density OIGT arrays with multimodal neuromorphic functions, especially when all active layers are patterned at high density [241]. Liu et al*.* [199] proposed a multimodal flexible OIGT array with different densities and combined it with an RC algorithm. By adopting an all-lithography method supported by a photocrosslinker, low variation and a high density of up to 9662 transistors cm^−2^ were achieved (Fig. 16c). The resulting OIGT array exhibits excellent neuromorphic performance, comparable to or even better than that of state-of-the-art IGT-based neuromorphic devices, while having the highest density. Five randomly selected regions (80 transistors) in the OIGT array achieved a 100% yield. For neuromorphic performance, the ion migration characteristics exhibited by the patterned ion gel layer endow the OIGT with good STP, such as EPSC/IPSC. These characteristics make the OIGT suitable for implementing the RC layer. In addition, by adding a large gate resistance, the OIGT can transition from a volatile state to a non-volatile state, enabling it to implement a physical readout layer (such as a multilayer perceptron network) to classify the RC results. The characteristics of long-term potentiation/depression, including nonlinearity (NL) and the dynamic range (*G*_max_/*G*_min_), were then systematically studied. Even when the unencapsulated flexible OIGT array was bent 1000 times to a radius of 1 mm or placed in air for 3 months, the above behaviors did not significantly degrade. Using the decaying memory of the flexible array for information compression and combining it with a software-based nonlinear neural network for back-end classification, the array achieved an accuracy of 97.21% in handwriting digit recognition, comparable to that of a traditional artificial neural network (97.19%) system, while significantly reducing the computational cost. In addition, using the multimodal operation ability of the flexible OIGT array, a flexible homogeneous RC system was theoretically constructed with an accuracy of 88.49%. The training accuracy achieved in this study is comparable to that of cutting-edge organic transistor neuromorphic devices. This work provides a promising OIGT array and RC algorithm design for future flexible artificial intelligence applications.

In addition to lithography-based patterning, various solution-processable and mechanically compliant fabrication approaches have been extensively explored for large-area integration of active layer materials in organic photonic synapses. Representative scalable techniques include inkjet printing [114, 242], spray coating [243, 244], and roll-to-roll printing [245, 246], which enable mask-free patterning, low-temperature processing, and compatibility with soft substrates. Soft lithography [247, 248] and nanoimprinting [249, 250] also provide high-resolution pattern transfer with reduced fabrication complexity. Indeed, as the field of photonic synapses, particularly organic photonic synapses, is still in its early stage of development, some fabrication technologies that are already well-established in other research areas such as perovskite solar cells have not yet been widely applied or reported in OPT-based synaptic systems. Nevertheless, it is foreseeable that with continued progress in large-area processing of functional layers and electrode fabrication techniques, the scalability and integration level of organic photonic synapses will be significantly advanced in the near future. These methods not only facilitate dense integration of synaptic arrays but also support conformable and wearable vision architectures, further highlighting the feasibility of artificial retinas based on organic phototransistors.

Beyond the above discussion, recent progress has demonstrated that photonic synapses can be integrated with additional sensory modalities such as chemical receptors [251], pressure sensors [252] and biological ion channels [178], enabling unified processing of heterogeneous stimuli [253]. Such multimodal platforms not only improve sensory selectivity, but also allow direct interface with neural tissues, paving the way for future bio-hybrid systems with advanced perception such as artificial olfaction [254], touch-assisted vision and neural-interfaced prosthetics [255]. These expansions illustrate the evolutionary direction of OPT-based synapses from vision-only systems toward comprehensive artificial sensory intelligence.

## Summary and Outlook

Overall, this review first summarizes the active organic semiconductor materials used in photonic synapses based on OPTs in recent years. Many materials are directly used as photosensitive layers to convert optical signals into electrical signals, whereas others serve as channel materials for separating and transporting photogenerated charges. Heterojunction structures that combine these two functions have become a popular choice for scientists to construct high-performance photonic synapses. In such structures, researchers have integrated perovskites, quantum dots, nanofibers, single-walled carbon nanotubes, and other nanostructures with high-performance OSCs to achieve low-energy-consuming and superior photonic synapses. By constructing heterojunction channel layers using p-type and n-type semiconductors to enable bidirectional transport of electrons and holes, persistent NPC has also been developed in OPT-based photonic synapses through gate voltage and light modulation. Benefiting from advancements in integration processes in recent years, diverse applications of photonic synapses have emerged, including basic pattern recognition functions such as mobile object recognition, handwritten digit recognition, and image recognition. On this basis, multifunctional photonic synapses integrating sensing, memory, and computing have been developed, enabling capabilities such as Morse code sensing, visual adaptation, night imaging, high-dimensional storage, polarization sensitivity, fused imaging, and on-sensor computing. Table 2 summarizes the device structures, fabrication processes, functionalities, and applications of the representative works discussed above.

**Table 2:** A summary of recent OPTs-based photonic synapses

Device	Fabrication methods	Device performance			Application	References
		Wavelength (nm)	Synaptic function	Special properties		
C8-BTBT/PTCDI-C_8_	Spin-coating and Thermal evaporation	405, 520	EPSC, PPF STP-to-LTP	Obstacle avoidance function	Mobile recognition	[75]
IR-780/PMMA/Pentance	Spin-coating and Thermal evaporation	880	EPSC, PPF STP-to-LTP	Artificial vision in NIR band	Handwritten digit recognition	[76]
CYTOP/DNTT/TPBi	Spin-coating	400–1000	EPSC, PPF	A recognition rate of over 70% across all wavelength bands	Face recognition	[77]
PU fiber/PEDOT:PSS/ZnO	Spin-coating	365	EPSC, PPF STP-to-LTP	Self-driven fibrous synapse	Pattern memorization and recognition	[128]
N2200/P0FD IID/PVA/IDTBT	Spin-coating	White Light	EPSC, IPSC	Feedforward photoadaptive	Robustness recognition	[129]
CsPbBr_3_QDs/C8-BTBT	Spin-coating and Thermal evaporation	365	EPSC, PPF STP-to-LTP	Energy consumption: 0.11 fJ	Morse cipher	[99]
PDPP-TT/CsPbBr_3_	Spin-coating	365	PPF, STP, LTP	Response speed < 35 ms	Visual adaptation	[100]
Ca_2_Nb_3_O_10_/PM_6_:PC 71BM	Spin-coating	300	EPSC, PPF	Photodetection and photosynapse	Imaging-to-recognition conversion	[101]
P3HT/CsPbBr_3_ QD	Spin-coating	405	EPSC, PPF STP-to-LTP	Energy consumption: 0.18 fJ	Memory and learning functionalities	[104]
PVCn:Pbs/C8-BTBT	Spin-coating	White Light	EPSC	Detecting scotopic-level illumination: 0.001 lx	Nighttime low illumination imaging	[105]
PI/AlO_x_/F8T2-SWCNT	Thermal evaporation, Lift-off processes and Atomic layer deposition	365, 450, 532, 650	EPSC, PPF STP-to-LTP	Energy consumption: 59 aJ	Image recognition	[112]
UCNPs@SiO_2_/P3HT	Spin-coating	980	EPSC, PPF STP-to-LTP	Infrared machine vision	High dimensionality reservoir expression	[113]
PPy-NPs	Ink-injecting	365, 455, 530, 590, 625, 680, 970	EPSC, PPF	Self-driven retinomorphic eye	Imaging and motion tracking	[114]
InGaAs NWs/C8-BTBT or PC_61_BM	Contact printing, Spin-coating	405, 450	EPSC, IPSC, PPF, STP-to-LTP	NPC, 100% accuracy	Hardware kernel	[169]
PTCDI-C_8_/C8-BTBT/ PTCDI-C_8_	Spin-coating and Thermal evaporation	520	EPSC, IPSC, PPF, STP-to-LTP	NPC and PPC in one device	Visual adaptation	[174]
(MBA)_2_PbI_2.8_Br_1.2_/PMMA/Pentacene	Spin-coating and Thermal evaporation	488	EPSC	Visible-light chiral photonic synapses	Circularly polarized system	[196]
PMMA/Ph-BTBT-10	Molecular self-assembly	365	EPSC, PPF STP-to-LTP	2D molecular crystal synapse	Polarization-sensitive visual system	[195]
Ag nanocluster crystals/Pentacene	Spin-coating	365, 473, 532, 633	EPSC	Vision hardware	Circular polarization vision	[219]
GaAs NWs/P3HT	Spin-coating, Molecular self-assembly	450, 532, 635	EPSC, IPSC	Birdlike broadband neuromorphic visual arrays	Information extraction, fusion visual imaging	[197]
TiO_2-x_ NRs/Pentacene/C_60_	Oblique angle deposition, Spin-coating and Thermal evaporation	400, 500, 600, 700, 800	EPSC, PPF STP-to-LTP	Dual mode devices	Binary security keys	[224]
Not specifically mentioned	Not specifically mentioned	395, 450, 640	EPSC, PPF STP-to-LTP	Record-high PPF	Biomimetic ocular system	[225]
PQD-nanocell-POSC	Four-step photolithography, Spin-coating and Thermal evaporation	Not mentioned	EPSC, PPF	Large-scale integration	Imaging recognition	[200]
In_2_O_3_/Ni-Au/PTB7-Th:Y6	Atomic layer deposition, Spin-coating	365, 808	EPSC, IPSC	Active-matrix synapse array	In-sensor spectral processing	[198]
DPPDTT/Ion-gel	Spin-coating and photolithography	Not mentioned	EPSC, PPF	All-photolithography fabrication	Multimode neuromorphic computing	[199]

Although OPT-based photonic synapses have achieved notable advancements in the aforementioned aspects, their practical applications still face significant challenges in terms of their molecular structure, operation behaviors/mechanisms, fabrication processes, integration, and system-level implementation. Specifically:

1. Molecular structure: Current research on organic photonic synapses has focused primarily on device engineering, while the relationship between molecular structure and synaptic performance has not been investigated in depth [256, 257]. The correlations between optoelectronic properties, such as charge transport rates, light absorption bands, and dielectric constants, and synaptic performance remain poorly understood.
2. Photoresponse behaviors/mechanisms: Current research on NPC photonic synapses remains relatively limited. Photonic synapses with gate voltage-modulated PPC/NPC exhibit imbalanced current levels. Moreover, owing to the uncontrollable nature of the external light intensity, light intensity-modulated PPC/NPC devices also face practical limitations in real-world applications.
3. From a practical application perspective, the simulation of photonic synapses should be more comprehensive. Most reported devices currently have relatively single functions, making it difficult to achieve functional integration. For example, photonic synapses capable of large-area lithography can respond only to specific forms of external light, whereas organic photonic synapses that can detect special light forms (such as polarized light) are challenging to fabricate via large-area lithography. Therefore, the integration level of multifunctional processing systems needs to be further improved [258–260]. Additionally, for applications simulating visual systems, photonic synaptic devices responsive to only a single wavelength or narrow wavelength range are insufficient. The inability to recognize and regulate light across different wavelengths limits the development of neuromorphic visual systems.
4. The existing fabrication methods: The vacuum evaporation method for film formation incurs high costs, is not suitable for roll-to-roll processes, and is difficult to scale up for mass production. On the other hand, the spin-coating process makes it difficult to achieve the patterned fabrication of organic semiconductor thin films directly and to define the channel dimensions of transistors. Moreover, neither of the above two fabrication schemes can meet the requirements of flexible substrates, which are more suitable for practical applications [261–263].

In summary, research on OPT-based photonic synapses is currently in a relatively mature stage, with diverse material systems and applications being extensively explored. However, current successes remain at the laboratory scale. If this field is to move toward practical applications, several key challenges must be addressed: improving environmental stability, optimizing fabrication processes, and achieving multifunctional integration. Specifically:

1. Application environments: Real-world scenarios are harsher than laboratory settings and involve uncontrollable high and low temperatures, among other factors. Additionally, owing to the inherent properties of organic materials—such as their sensitivity to water and oxygen—they often exhibit significant performance degradation in a short time compared with the high stability of inorganic materials, which hinders the realization of stable photostimulated synaptic processes [264–267]. In reality, the human eye is surrounded by a watery environment, making the development of corresponding encapsulation processes essential.
2. Fabrication processes: The manufacturing of photonic synapses largely depends on the functional semiconductor materials and device structures employed. In laboratories, organic photonic synapses can be conveniently prepared via techniques such as thermal evaporation and spin-coating to improve fabrication efficiency and optimize device performance. However, for practical applications such as commercial production—especially those requiring large-area fabrication or processing on flexible substrates—methods such as inkjet printing [268–270] and 3D synchronous multimaterial printing [271–273] have become necessary. The inkjet printing technology developed by Chen et al. [274] has established a viable approach for fabricating multifunctional synaptic arrays on large-area flexible substrates. Composite encapsulation can be achieved through atomic layer deposition (ALD) [275, 276] or chemical vapor deposition (CVD) [277, 278] to isolate oxygen and water with multilayer thin films. For instance, Yang et al. [279] have demonstrated the potential of ALD technology for encapsulating organic-inorganic hybrid materials, showing promising results in enhancing device stability. When other fabrication processes, such as lift-off processes or molecular self-assembly [264, 280–282], are developed, it is also essential to address obstacles that limit the scalable application of neuromorphic devices.
3. Integration and System Implementation: Future development of photonic synapses should address current limitations in gate-voltage-dependent and light-intensity-dependent NPC devices by designing complementary PPC/NPC synaptic systems with balanced wavelength regulation and optimized material thickness. Beyond memory functions, ideal photonic synapses should incorporate optical signal erasure capabilities. The integration of photosynapses with photodetectors enables high-quality optical signal acquisition and perception [101]. Furthermore, by incorporating functional materials such as COF films [266, 267] for photo/gas-actuated responses or triboelectric nanogenerators [283, 284] for piezoelectric conversion, intelligent photonic synapses capable of sensing temperature, humidity, gas, and pressure can be developed [260], advancing applications in environmental monitoring and flexible robotics. At the system level, successful implementation requires architectural strategies beyond single-device engineering. Hardware partitioning—separating photonic sensing from plasticity modulation in interconnected units—enhances electrical stability and reduces crosstalk in large arrays [285–287]. Critical considerations also include peripheral circuitry optimization, interconnect design, and compensation for device-to-device variations. Advances in manufacturing, such as printing-assisted alignment [288] and transfer-based flexible integration [289], are accelerating the scaling of retina-like sensor networks with in-sensor computing and pattern recognition capabilities. Together, these approaches bridge the gap between laboratory prototypes and deployable neuromorphic vision systems.

## Abbreviations

ALD: Atomic layer deposition
ANN: Artificial neural network
AVN: Artificial visual neuron
AVS: Artificial visual system
BHJ: Bulk heterojunction
BBNVS: Birdlike broadband neuromorphic visual sensor
CD: Circular dichroism
CPL: Circularly polarized light
CMOS: Complementary metal–oxide–semiconductor
CNF: Composite nanofiber film
CNT: Carbon nanotube
CPU: Central processing unit
CVD: Chemical vapor deposition
D/A: Donor/acceptor
DPS: Dual-photo synapse
ECT: Electrochemical transistor
EDS: Energy-dispersive X-ray spectroscopy
EPSC: Excitatory postsynaptic current
ESFGT: Electrically stimulated floating-gate transistor
FET: Field-effect transistor
FPAS: Fiber photonic artificial synapse
HOIP: Hybrid organic–inorganic perovskite
IGZO: Indium gallium zinc oxide
IPSC: Inhibitory postsynaptic current
IPQD: Inorganic perovskite quantum dot
IR: Infrared
LCP: Left-circularly polarized
LDR: Linear dichroic ratio
LIF: Leaky integrate-and-fire
LSI: Large-scale integration
LTM: Long-term memory
LTP: Long-term plasticity
M-M: Metal-metal bonding
NIR: Near-infrared
NL: Nonlinearity
NPC: Negative photoconductance
NR: Nanorod
NW: Nanowire
OFET: Organic field-effect transistor
OIGT: Organic ion-gated transistor
OPT: Organic phototransistor
OSC: Organic semiconductor
OSAT: Organic scotopic adaptation transistor
P3HT: Poly(3-hexylthiophene)
PQD: Perovskite quantum dot
PET: Polyethylene terephthalate
PHD: Presynaptic homeostatic depression
PI: Polyimide
PL: Photoluminescence
PMMA: Poly(methyl methacrylate)
PPC: Positive photoconductance
PPD: Paired-pulse depression
PPF: Paired-pulse facilitation
PSC: Postsynaptic current
PS: Polystyrene
PUF: Physical unclonable function
PVCn: Poly(vinylcarbazole)
QD: Quantum dot
RC: Reservoir computing
RCP: Right-circularly polarized
sc-SWCNT: Semiconducting single-walled carbon nanotube
SMD: Stimulus-moment-dependent
SNN: Spiking neural network
STDP: Spike-timing-dependent plasticity
STM: Short-term memory
STP: Short-term plasticity
SW: Synaptic weight
TFT: Thin-film transistor
ULSI: Ultra-large-scale integration
UV: Ultraviolet
vdW: Van der Waals
VLSI: Very-large-scale integration
VO: Oxygen vacancy
WD: Wavelength-dependent
